# Sex-specific changes in the hippocampal proteome of *Negr1*^*−/−*^ mice: insight into the mechanisms of neuropsychiatric disorders

**DOI:** 10.1186/s13293-026-00890-0

**Published:** 2026-03-26

**Authors:** Srirathi Muthuraman, Mohan Jayaram, Liisi Promet, Toomas Jagomäe, Arun Kumar Devarajan, Andreas-Christian Hade, Mari-Anne Philips, Katyayani Singh, Eero Vasar

**Affiliations:** 1 Institute of Biomedicine and Translational Medicine, Department of Physiology, University of Tartu, Tartu, Estonia; 2 Institute of Molecular and Cell Biology, University of Tartu, Tartu, Estonia; 3 Department of Pathological Anatomy and Forensic Medicine, University of Tartu, 19 Ravila Street, Tartu, 50411 Estonia

**Keywords:** Negr1, Sex-difference, Neuropsychiatric disorder, Animal model, Proteomics, Sex-specific, Pvalb, PV, Hippocampus

## Abstract

**Background:**

Neuronal Growth Regulator 1 (NEGR1) is a cell adhesion molecule involved in hippocampal circuit development and function. Human genetic studies have identified NEGR1 variants as risk factors for a broad spectrum of neuropsychiatric disorders. These disorders often display sex-specific differences in prevalence, progression, and behavioral impairment, reflecting underlying maladaptive changes in neural circuitry. Findings from preclinical studies using *Negr1*^*−/−*^ mice show several hippocampal-based behavioral and anatomical endophenotypes relevant to neuropsychiatric disorders. The hippocampus, a key region implicated in these disorders, exhibits sex-dependent anatomical features that may shape the functional impact of Negr1. However, the mechanisms driving these sex-specific characteristics have not yet been elucidated. Here, we uncover sex-specific molecular signatures and pathways associated with Negr1, using *Negr1*^*−/−*^ mice, a genetically relevant animal model for neuropsychiatric risk.

**Methods:**

We performed label-free quantitative proteomic analysis using eight replicates of hippocampi dissected from male and female wild-type, and *Negr1*^*−/−*^ mice. Differentially abundant proteins were subjected to functional annotation for Gene Ontology and Protein-Protein interaction using STRING analysis. NEGR1 cellular localization was examined by immunofluorescent in rat brain and human hippocampal sections.

**Results:**

Differential expression analysis identified 232 proteins in males and 172 in females. STRING analysis revealed sex-specific regulation of proteins. In males, proteins linked to neurofilament organization, myelin integrity, and postsynaptic structure were downregulated, with parvalbumin (Pvalb, PV) around the central node. In contrast, proteins related to mitochondrial and stress-response pathways were upregulated. Female *Negr1*^*−/−*^ hippocampus showed downregulation of proteins involved in translation and amide biosynthetic processes. Colocalization of NEGR1 with PV interneurons in the rat brain and the human hippocampus was observed.

**Conclusions:**

We demonstrate, for the first time, distinct sex differences in the hippocampal proteome and identify molecular networks in *Negr1*^*−/−*^ mice. Co-localization of NEGR1 and PV in human brain tissue provides anatomical and translational validation of a proteomic target. These findings provide new insight, offering a valuable resource for understanding NEGR1-related sex-specific mechanisms in neuropsychiatric disorders.

**Supplementary Information:**

The online version contains supplementary material available at 10.1186/s13293-026-00890-0.

## Abstract (plain language summary)

The hippocampus is a key brain structure for learning, memory, and emotional regulation, and its dysfunction is central to several neuropsychiatric disorders, including major depression, schizophrenia, autism spectrum disorders, anxiety, and cognitive decline that are associated with Negr1 gene variants. The prevalence, expression, and course of neuropsychiatric disorders vary substantially between males and females.

Neuronal Growth Regulator 1 (Negr1) is a member of the IgLON family of cell adhesion molecules. Negr1 is involved in the formation of neuronal circuits during prenatal and postnatal development. Negr1 deletion (*Negr1*^*−/−*^) in mice causes several abnormalities in the hippocampus, which underlie behavioral changes like those observed in a neuropsychiatric disorder.

To understand the protein expression profile, protein-interaction network, and signaling pathways underlying Negr1-associated neurobehavioral pathology, we performed unbiased proteome analysis of the hippocampus from male and female *Negr1*^*−/−*^ mice, which has not been done previously. We identified distinct sex-specific, differentially abundant proteins and biological pathways that differ between male and female tissues. We found that NEGR1 is located together with a special type of calming brain cell, known as parvalbumin neurons in rat and human brain, suggesting that they may work closely together in the brain. These would provide important data to further investigate the underlying sex-specific neurobiology of behavioral impairments related to Negr1 pathology.

**Supplementary Information:**

The online version contains supplementary material available at 10.1186/s13293-026-00890-0.

## Highlights

- Sex-specific changes in the hippocampal proteome of Negr1-/- mice.
- Proteome analysis in male hippocampus highlights pathways related to cytoskeletal and myelin programs, along with PV interneuron associations.
- Female pathways feature mainly translation and proteostasis by limiting synaptic renewal.
- This exploratory work highlights the need to avoid generalizing male-derived evidence in future preclinical studies.
- This dataset will serve as a resource for researchers to explore sex specific pathways associated with Negr1 disease pathology.

**Supplementary Information:**

The online version contains supplementary material available at 10.1186/s13293-026-00890-0.

## Background

Sex and gender exert profound influence on molecular signaling and neural circuit dynamics, underpinning distinct patterns of vulnerability and resilience in neuropsychiatric disorders [1]. Genome-wide association studies (GWAS) have identified strong genetic correlations and widespread pleiotropy across these disorders, further obscuring insights into the underlying maladaptive neurocircuits [2–5]. Although sex differences in the prevalence of neuropsychiatric disorders are well documented, progress in improving treatment efficiency and addressing disparities in disease remains limited. The limited understanding of sex differences in disease mechanisms is partly a consequence of the long-standing exclusion of female rodents from preclinical studies [6, 7]. Consequently, key sex-dependent differences in neurocircuitry and molecular mechanisms remain poorly understood, limiting the development of sex-specific therapeutic strategies.

A wide range of multi-omics and clinical studies consistently identify neuronal growth regulator 1 (NEGR1) as a key gene implicated in neuropsychiatric, neurodegenerative, and metabolic disorders. NEGR1 is associated with several complex conditions, including major depressive disorder, schizophrenia, autism spectrum disorders, ADHD, intellectual disability, language impairment, Alzheimer’s disease, Parkinson’s disease, prion diseases, multiple sclerosis, Niemann–Pick type C disease, and metabolic traits such as body-mass regulation and obesity [8–11]. Preclinical findings from *Negr1*^*−/−*^mouse models further support these links, revealing endophenotypes that mirror human disease mechanisms across neuropsychiatric and comorbid conditions [12–17].

NEGR1 is present during early developmental stages in rodents, indicating its essential role in shaping neural circuits that support sensory processing, emotional regulation, and cognitive functions [18–20]. Notably, Negr1 is expressed in the evolutionarily conserved medial pallium—the histogenetic domain that forms the hippocampal structure—highlighting its importance in hippocampal development [19, 21]. The hippocampal formation comprises the hippocampus proper (CA1–CA3), the dentate gyrus, the subicular complex, and the entorhinal cortex. It serves as an integration hub for highly processed multimodal sensory information, underpins key cognitive and behavioral functions, including memory, spatial navigation, pattern recognition, social memory, and flexible sensory associations [22]. This makes it one of the most important brain regions for investigating the mechanisms underlying multispectral neuropsychiatric disorders [23–27].

A considerable number of hippocampal neurocircuitry aberrations have been identified in *Negr1*^*−/−*^ mice, encompassing neurodevelopmental processes such as cortical layering, neuronal migration, neuritogenesis, neurite outgrowth, dendritic spine density, and synaptic connectivity [13, 28–30]. In adulthood, *Negr1*^*−/−*^ mice exhibit impairments in inhibitory neurotransmission, including reduced parvalbumin (PV/Pvalb)-positive interneurons and abnormal synaptic GABA synthesis [13, 31]. These mice also show impaired axonal growth within the hippocampal–entorhinal circuit, deficits in dentate gyrus long-term potentiation, reduced neurogenesis in the olfactory system and hippocampus, decreased hippocampal and overall brain volume with ventricular enlargement [12–14, 17]. Such alterations disrupt both intrahippocampal connectivity and their connections with other brain areas, which are critical for cognition, emotion, social, and sensory processing, thought to underlie the broad behavioral deficits observed in *Negr1*^*−/−*^ mice [25, 27, 32].

A growing body of evidence across humans and rodent models indicates that the hippocampal morphology, connectivity, physiological properties, and molecular mechanisms are distinctively organized between males and females [33, 34]. The molecular mechanisms driving sexually dimorphic changes in the hippocampus, including those involving *Negr1*, are not yet understood. Understanding these mechanisms is critical for developing sex-specific diagnostic and therapeutic strategies for complex disorders related to NEGR1. Therefore, here we aim to characterize baseline sex-specific differences in hippocampal protein composition in male and female *Negr1*^*−/−*^ mice by applying an untargeted quantitative proteomics approach. This will provide insights into the underlying biological mechanisms that differ between males and females, informing a sex-specific understanding of disease.

## Materials and methods

### Animals

Male and female wild-type (Wt) mice and their homozygous *Negr1*-deficient littermates (*Negr1*^*−/−*^, KO), which were previously described [35] in F2 background ((129S5/SvEvBrd × C57BL/6 N) × (129S5/SvEvBrd × C57BL/6 N)) were used for Proteomics analysis. The estrous cycle stages were not monitored in female mice.

Male (Wt and KO) and female (Wt and KO), all four groups of mice were housed (in groups of 8–10) separately in normal laboratory cages measuring 42.5 (L) × 26.6 (W) × 15.5 (H) cm. Males’ cages were kept in different racks in the same room, from weaning until the completion of the experiments. The cages were kept at 22 ± 1 °C with a 12:12 h light/dark cycle (lights went off at 19:00 h). Each cage had a 2 cm layer of aspen bedding (Tapvei, Estonia) and 0.5 l of aspen nesting material (Tapvei, Estonia), both of which were replaced once a week. Food pellets (R70, Lactamin AB, Sweden) and water (purified via reverse osmosis) were provided ad libitum.

Wt Sprague Dawley rats (Crl: CD(SD) IGS, Charles River Laboratories) were used for immunohistochemical analysis. Rats were housed in groups of 2–4 under standard conditions with a 12-hour light/dark cycle (lights on at 7:00 AM), and had ad libitum access to food (Sniff universal mouse and rat diet, Ssniff #V1534) and water (purified via reverse osmosis).

The mice and rats were bred and maintained at the Institute of Biomedicine and Translational Medicine’s animal facility at the University of Tartu in Estonia. Regulations and guidelines authorized by the Laboratory Animal Centre at the Institute of Biomedicine and Translational Medicine, University of Tartu, Estonia, were followed when handling mice. According to the European Communities Directive (2010/63/EU), animal experiments were conducted with approval from the Estonian National Board of Animal Experiments, Põllumajandus- ja Toiduameti (No. 150, 27 September 2019). As stated at https://arriveguidelines.org, we certify that this work is reported in compliance with the ARRIVE (Animal Research: Reporting of In Vivo Experiments) guidelines.

### Human brain tissue

Postmortem hippocampal samples were obtained from clinically healthy individuals (*n* = 5, mean age 31 ± 8.4 years; 3 males, 2 females) by the Department of Pathological Anatomy and Forensic Medicine, University of Tartu, and the Estonian Forensic Science Institute. Comprehensive details of the human sample characteristics, including sex and age, are presented in Supplementary Material 1 (Supplementary Table 1). All procedures followed ethical approval from the University of Tartu Human Research Ethics Committee (223/T-4). Human brains were perfused and stained according to the protocol described in [36].

### Proteomics analysis

#### Sample preparation

Hippocampal tissues from 7-month-old male and female *Negr1*^*−/−*^ and Wt mice were collected and immediately snap-frozen until further processing. Eight Wt and eight *Negr1*^*−/−*^ animals were used from male mice, and similarly, eight *Negr1*^*−/−*^ and eight Wt animals were used from female mice. In total, the study included 16 males and 16 females, comprising 32 animals overall. We aimed to adopt an unbiased experimental approach by studying animals under normal housing conditions, without controlling for estrous stage, to establish baseline phenotypic differences.

Proteins were extracted from the hippocampal tissue using a lysis buffer containing 6 M guanidine hydrochloride (Gu-HCl) and 100 mM Tris (pH 7.5). The lysates were sonicated, vortexed, and centrifuged to remove debris. The supernatant containing solubilized proteins was carefully transferred to fresh tubes. Protein concentration was measured using the Thermo Fisher Scientific Micro-BCA Protein Assay Kit (Thermo Fisher Scientific − 23235), following the manufacturer’s protocol. Standards and samples were incubated at 65 °C for 15 min, and absorbance was read at 562 nm with a reference wavelength of 700 nm. Protein concentrations were determined using a standard curve generated from BSA standards.

Following quantification, equal amounts of protein were subjected to in-solution digestion. Proteins were reduced, alkylated, and digested overnight at 37 °C with sequencing-grade modified trypsin. No additional proteases were used. The digestion protocol was carried out by established procedures at the Tartu University Institute of Technology Proteomics Core Facility (TUIT PCF). Post-digestion, peptides were desalted using C18 solid-phase extraction tips (StageTips) and dried using a SpeedVac. Peptides were not labeled isotopically.

### Protein quantification and identification

Label-free quantification (LFQ) was performed using a Q Exactive Plus (Thermo Fisher Scientific, Waltham, MA, USA) tandem mass spectrometer combined with Dionex nano-RSLAC 3500 nano-LC. Raw MS data were processed using MaxQuant software (v2.6.7.0) with default parameters for LFQ, enabling the ‘Match between runs’ function. Protein identification was performed against the UniProt mouse reference proteome (UP000000589). Quality filtering retained only proteins identified with at least one unique peptide and a false discovery rate (FDR) < 1%. The resulting Protein Groups output file was exported for downstream statistical analysis. All statistical analyses were performed using R packages.

### Quantification of differential protein abundance and Statistics

Differential abundance (DA) analysis of identified proteins was conducted using the ProDA package (https://const-ae.g.ithub.io/proDA/), which applies linear modeling with Bayesian priors to enhance statistical power. Data was preprocessed before DA analysis. A presence threshold of six was applied in at least one sample group. Missing values were imputed separately within each group using a random uniform distribution, ranging from 90% to 110% of the minimum observed non-zero value, to ensure a conservative approximation without inflating expression levels. The Benjamini–Hochberg procedure was used to control the FDR in DA analysis in the ProDA package [37]. Proteins with *p* < 0.05 were considered significantly differentially abundant. Finally, it was visualized as a volcano plot using the ggplot2 package. A Venn Diagram was constructed using the ggvenn package in R [38] to visualize and compare total and significantly expressed proteins identified in male and female samples from both thresholds. Principal Component Analysis (PCA) was performed using the prcomp function in the stats package, and the results were visualized with ggplot2 to assess sample variation across treatments. The effect of treatment on overall protein abundance profiles was evaluated by PERMANOVA analysis using the adonis2 function in the vegan package.

### Enrichment analysis

Functional annotation of DA proteins was performed using STRING, with enrichment analyses for GO (Biological Process, Molecular Function, Cellular Component, Reactome, and KEGG pathway). GO and Protein-Protein interaction (PPI) analysis for differentially expressed targets was performed using STRING (https://string-db.org/). The PPI Network was constructed from STRING database interactions with high confidence (combined score > 0.6), clustered using the Markov Clustering Algorithm (MCL) with an inflation parameter of 2.0 to identify functional modules. The igraph package in R and the Fruchterman-Reingold layout were used to organize the network spatially for visualization [39]. Proteins were labeled by their names, and nodes were colored according to their cluster assignment using a colorblind-friendly palette (Set2 from hcl.colors) using ggplot2.

### Immunohistochemical analysis

#### Immunohistochemistry of rat brain tissue

Adult rat brains were perfused as follows: rats were anesthetized via intraperitoneal injection of ketamine/dexmedetomidine in physiological saline (150 mg/kg ketamine, 0.5 mg/kg dexmedetomidine; 0.1 mL/100 g body weight). Upon reaching deep anesthesia, the thoracic cavity was opened, and perfusion was performed by inserting a needle into the left ventricle and making an incision in the right atrium. Blood was flushed with phosphate-buffered saline (PBS; 50 mL/min), followed by fixation with 4% paraformaldehyde in PBS (50 mL/min).

Perfused and fixed brains were cryoprotected in 30% sucrose in PBS and frozen at -80 °C. Coronal, sagittal, and horizontal 30 μm cryosections were obtained using a Leica CM1520 cryostat and stored in PBS. Sections were washed (3 × 5 min in PBS), permeabilized for 45 min in 0.25% Triton X-100 in PBS, and blocked overnight in a solution containing 0.3 M glycine, 5% normal goat serum (NGS), 1% bovine serum albumin (BSA), and 0.1% Tween-20 in PBS.

Sections were incubated for 48 h at 4 °C in primary antibodies: rabbit anti-NEGR1 (0.5 µg/mL, Sigma-Aldrich, HPA011894) and guinea pig anti-parvalbumin (1:250, Synaptic Systems, 195 004). After washing, sections were incubated for 2 h at room temperature in secondary antibodies: Alexa Fluor 488-conjugated goat anti-rabbit (3 µg/mL, Jackson ImmunoResearch 111-545-045) and TRITC-conjugated donkey anti-guinea pig (3 µg/mL, Jackson ImmunoResearch 706-025-148). DNA was counterstained using Hoechst 33,258 (5 µg/mL, 10 min), and sections were mounted with 0.5% gelatin and Fluoromount. Coverslips (0.13–0.16 mm) were applied. Negative controls (omitting primary antibodies) were used to confirm antibody specificity. Imaging was performed using a Leica SCN400 slide scanner; panels were compiled with GIMP v2.10.22. Brain regions were identified using the rat brain atlas [40].

To assess the colocalization of NEGR1 expression in PV+ cells within the rat hippocampus, 30 μm sections were collected every 600 μm from Bregma coordinates − 1.72 to − 6.84 mm (coronal) and − 3.10 to − 9.10 mm (horizontal). After immunohistochemistry, hippocampal subregions (subiculum, CA1–CA3, dentate gyrus) were outlined, and cells expressing PV only or both PV and NEGR1 were counted using Image J.

#### Immunohistochemistry of human hippocampus

For immunohistochemistry analysis, the autopsy human brain was perfused using our previously published ex vivo whole-brain perfusion protocol without modification [36]. Immunohistochemistry of human hippocampal tissue was performed using a modified protocol [41]. The dissected tissue was cryoprotected in 30% sucrose in PBS for 4 days and then frozen at − 80 °C. Coronal Sects. (30–40 μm) were obtained using a Leica CM1520 cryostat. Sections were permeabilized in 0.2% Triton X-100 (1 h to overnight) and subjected to antigen retrieval in 0.1 M sodium citrate buffer (pH 4.5) at 95 °C (3 × 5 min).

Sections were blocked for 1 h in 0.3 M glycine, 5% NGS, 1% BSA, 0.2% Triton X-100 in PBS, followed by 4-day incubation in primary antibodies (same as for rat tissue) diluted in 1% NGS and 0.2% Triton X-100 in PBS. After washing, sections were incubated overnight in secondary antibodies (same as above) in 1% NGS, 0.2% Triton X-100 in PBS. Autofluorescence was quenched by incubating with 1% Sudan Black in 70% ethanol for 40 min. Nuclear staining was performed using Hoechst 33,258 (5 µg/mL, 10 min). Mounting and imaging protocols matched those used for rat brain sections. The specificity of the secondary antibody was confirmed using negative controls. Human hippocampal subregions were identified using the human brain atlas [42].

## Results

Here, we performed proteomic analysis using mouse hippocampal tissue and the translational relevance of NEGR1 localization findings through immunostainings in rat and human postmortem brain samples.

### Hippocampal proteomic profiling of male and female *Negr1*^*−/ −*^mice

Quantitative proteomic analysis was performed using 8 replicates of hippocampi dissected from Wt and *Negr1*^*−/−*^ in male and female mice at 7 months of age. Seven-month-old mice were selected because this age reliably captures the emergence of behavioral, metabolic, and MRI-defined anatomical alterations in *Negr1*^*−/−*^ animals, as demonstrated in our previous studies [12, 13, 15, 20]. This time point, therefore, enables the investigation of key mechanistic pathways underlying endophenotypes related to neuropsychiatric disorders. Body weight was monitored once in a month from 2 to 3 months of age. No significant differences were detected in males; however, female mice showed similar weight differences that emerged at 4–5 months of age, as previously reported [13].

**Fig. 1:**
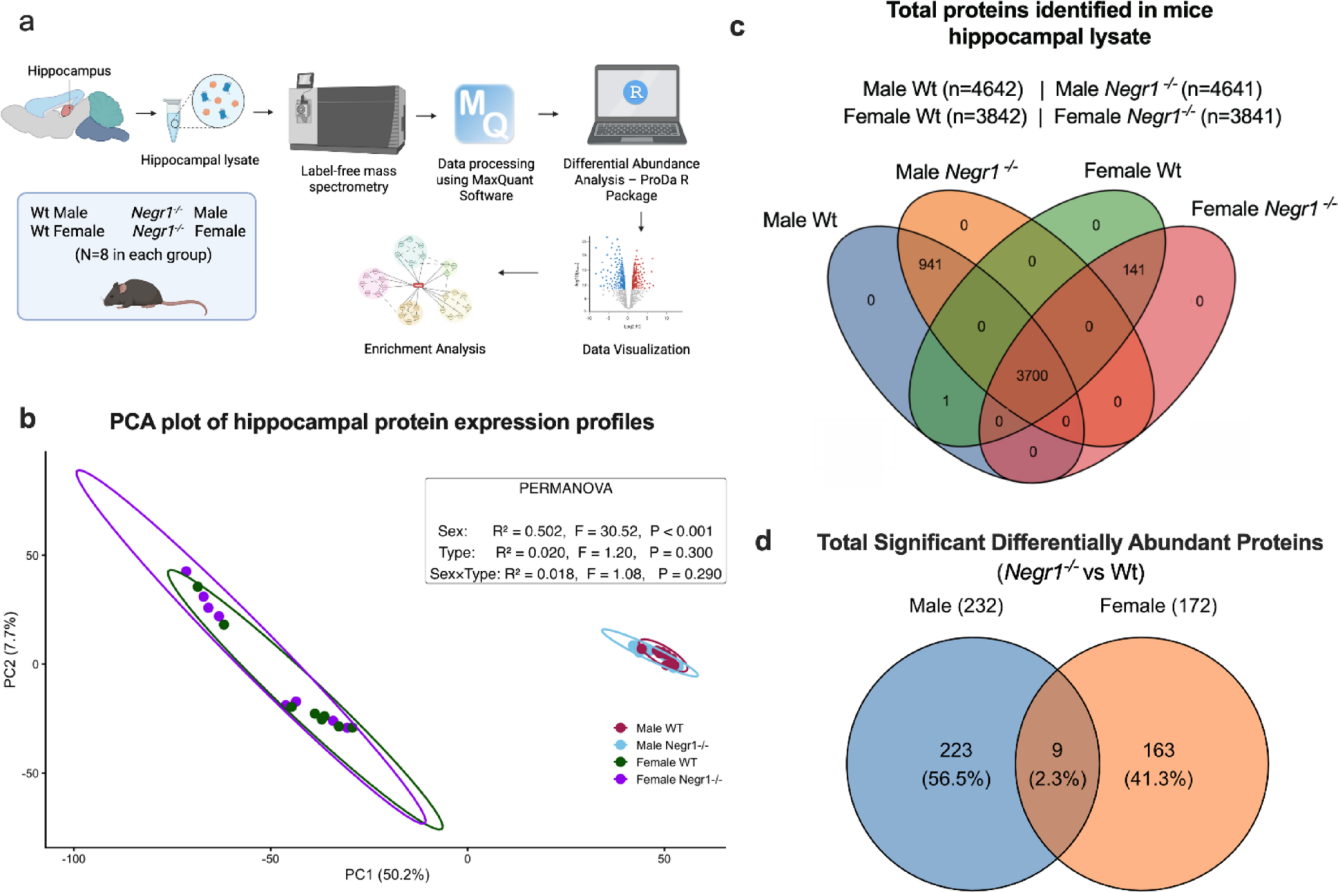
Experimental workflow for Hippocampal proteome analysis, Total proteins and principal component analysis (PCA) of hippocampal proteomes in wild-type (Wt) and *Negr1* KO (*Negr1*^*−/−*^) mice. (**a**) Schematic overview of the experimental design. (**b**) PCA plot of hippocampal protein expression profiles. Each point represents an individual mouse (male WT: dark red; male *Negr1*^*−/−*^: light blue; female WT: dark green; female *Negr1*^*−/−*^: purple). (**c**) Venn diagram of the total number of proteins identified in male and female samples in both Wt and *Negr1*^*−/−*^, showing the unique and common proteins. (**d**) Venn diagram illustrating the unique and common differentially abundant proteins in male and female samples (*Negr1*^*−/−*^ vs. Wt)

Proteins were analysed using LC/MS MS-based LFQ, the experimental workflow is illustrated in Fig. 1a. The first two principal components explain 50.2% (PC1) and 7.7% (PC2) of the total variance Fig. 1b. The PERMANOVA analysis indicates that sex explains 50.2% of the variance in the hippocampal proteome and is statistically significant (R² = 0.502, *p* < 0.001), showing a strong separation between male and female samples. In contrast, genotype explains 2.0% of the variance (R² = 0.020, *p* = 0.300) and the sex × genotype interaction explains 1.8% of the variance (R² = 0.018, *p* = 0.290); neither effect is statistically significant. Despite the modest contribution of genotype to global proteomic variance, subsequent differential abundance analyses identified specific protein alterations associated with Negr1 deficiency. This indicates that *Negr1* knockdown did not cause major alterations in the global proteome composition. Because our mice have an F2 mixed genetic background (129S5/SvEvBrd × C57BL/6 N), the lack of distinct clustering among experimental groups was expected and reflects natural genetic variability. Therefore, we checked LFQ intensities in both male and female *Negr1⁻/⁻* samples, and the absence of LFQ signals confirmed that Negr1 protein was undetectable in these specimens. Label-free mass spectrometry identified a total of 4,642 proteins in male WT, 4,641 in male *Negr1*^*−/−*^, 3,842 in female WT, and 3,841 in female *Negr1⁻/⁻* hippocampal lysates. Venn diagrams depict the total number of identified proteins (Fig. 1c), unique and common differentially abundant proteins (Fig. 1d**)** in male and female samples (*Negr1*^*−/−*^ vs. Wt). The male and female significantly regulated proteins were compared with the male and female total proteins to obtain the percentage mentioned. Negr1 is the one protein that is absent in both male and female *Negr1*^*−/−*^ mice. Across all four groups, 3,700 proteins were commonly identified regardless of sex or genotype. Separate venn diagrams showing total number of protein identified in male and female Wt and *Negr1* ^*−/−*^ hippocampal lysate, were found in Supplementary Fig. 1a-b.

DA analysis revealed that only 5.0% and 4.5% of proteins in male and female samples, respectively, were significantly altered across groups. Specifically, in male samples, 232 proteins (65 upregulated and 167 downregulated) were differentially abundant in *Negr1*^*−/−*^, and in female samples, 172 proteins (37 upregulated and 135 downregulated) were differentially abundant in *Negr1⁻/⁻* mice. Out of these, 9 proteins were found to be common DA proteins that are present in both males and females, including *Bcat2*, *Ca2*, *Car2*, *Ermn*, *Ero1l*, *Fam45a*, *Lsm12*, *Nanp*, *Sf1*, *and Snx32. Lsm12* is downregulated in both males and upregulated in females; details are found in (Supplementary Material 1; Table S1.1, S1.2 and in Supplementary Material 2; Table S1*)*. We included proteins identified by at least one unique peptide, as low-abundance proteins are sometimes detectable only through a single unique peptide. This approach has been adopted in recent proteomics studies [43, 44], where single-peptide identifications were accepted under stringent quality control criteria. To enhance data reliability, we applied a minimum presence threshold of six valid values per group to reduce the impact of missing data. Missing values were conservatively imputed within each group using a narrow uniform distribution (90–110% of the minimum non-zero intensity) to avoid artificial inflation of protein abundance.

**Fig. 2:**
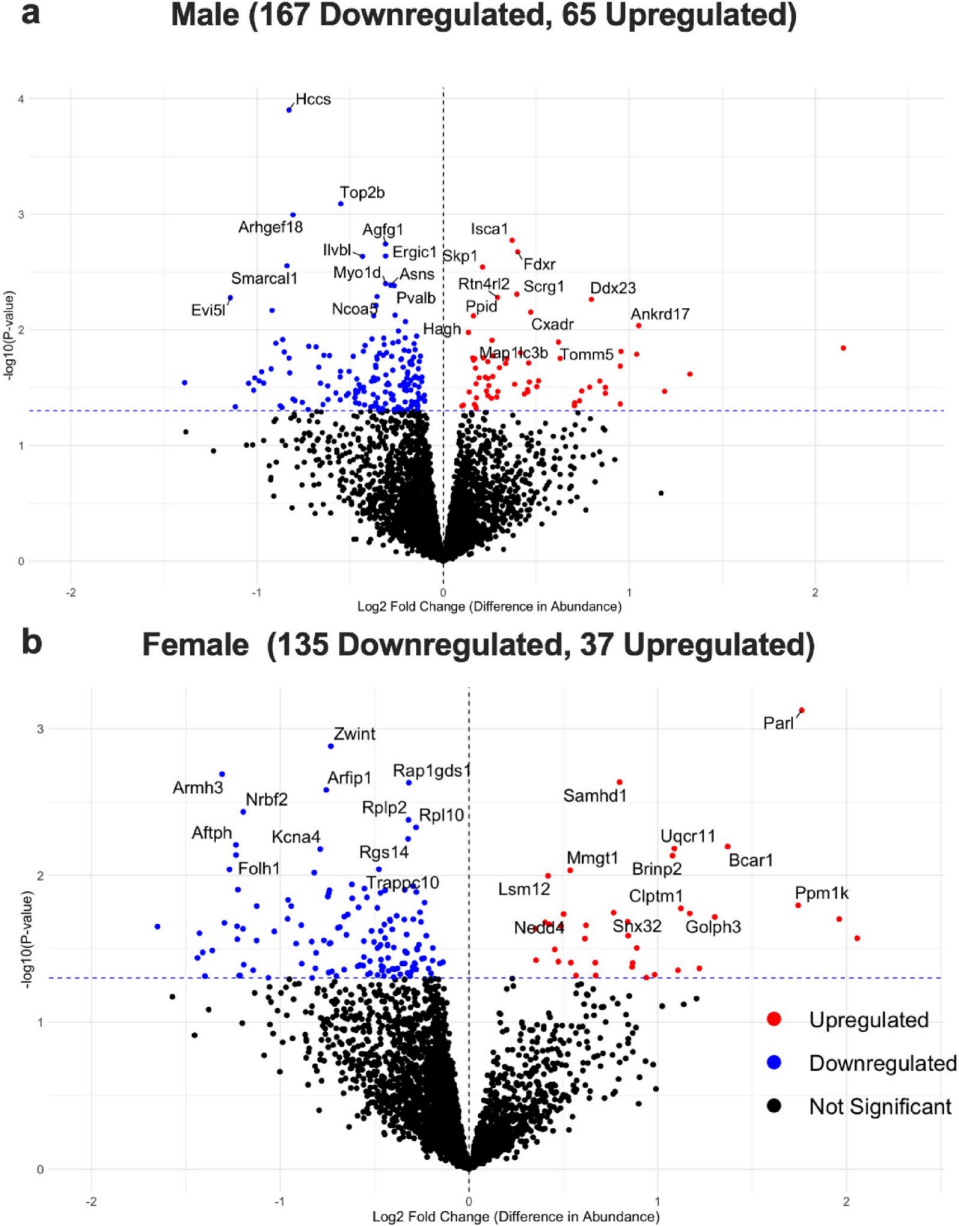
Differential Abundance of Proteins between Wt and *Negr1*^*−/−*^ in both male and female samples (**a**) Volcano plot depicting differentially expressed proteins in male samples, with red indicating upregulated and blue indicating downregulated proteins. (**b**) Volcano plot showing differentially expressed proteins in female samples, with red indicating upregulated and blue indicating downregulated proteins. The top 12 upregulated and downregulated proteins were labeled based on their p-value

The DA proteins were illustrated in the volcano plots for male and female *Negr1*^−/−^ hippocampal issues separately in Fig. 2a-b and details of all the significant proteins are listed in Supplementary Material 2 (Tables S2-S5).

### Identification of enriched pathways

**Fig. 3:**
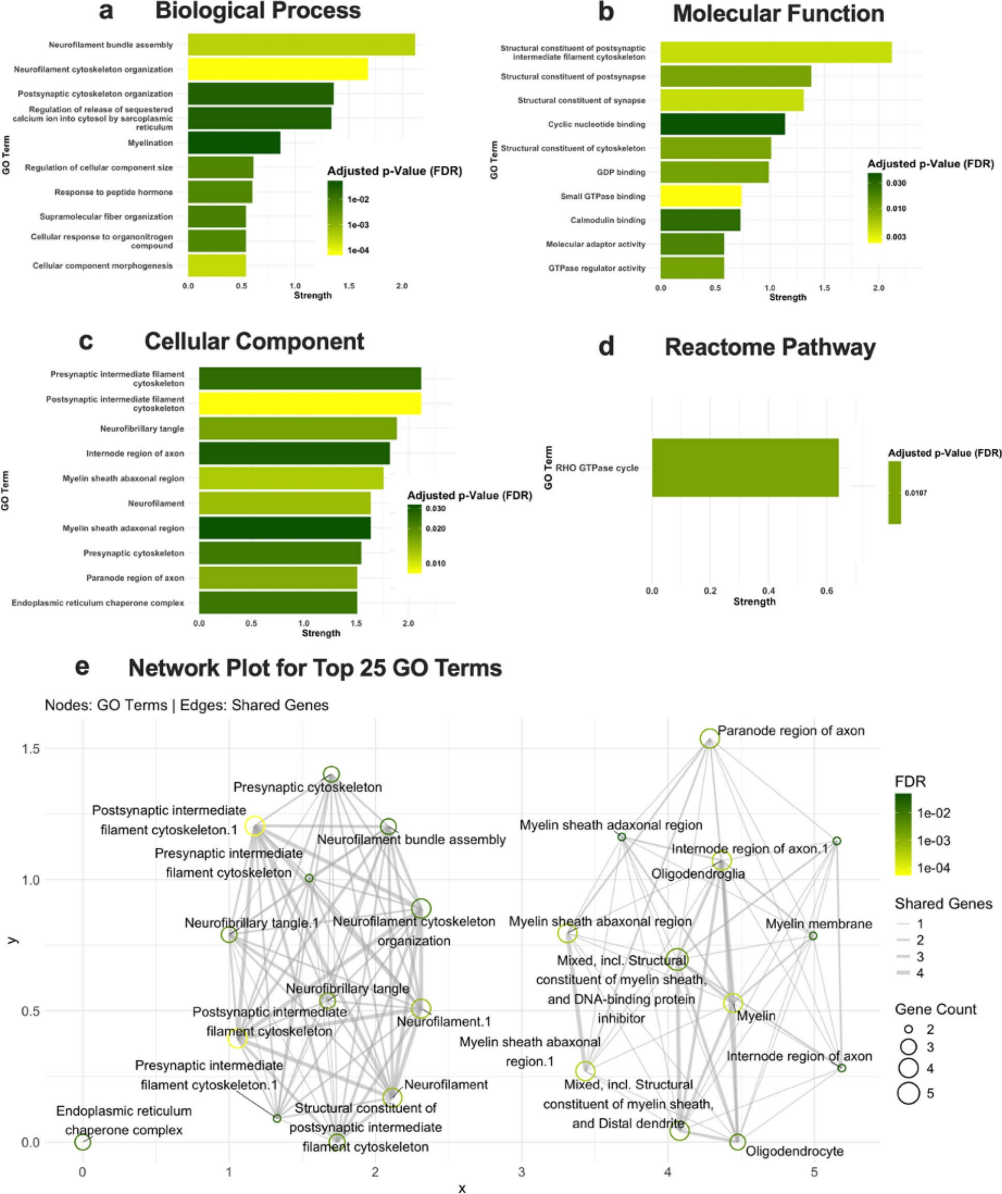
Male Gene Ontology (GO) with downregulated proteins: Enrichment Analysis for significantly downregulated proteins (p-value < 0.05) in male *Negr1*^*−/−*^ mice using STRING analysis. GO term enrichment for (**a**) Biological Process, (**b**) Molecular Function, (**c**) Cellular Component, and (**d**) Reactome Pathway. Bar plots show the top enriched GO terms, with the x-axis representing enrichment strength, and bar color indicates the FDR (false discovery rate). (**e**) displays a network plot of the top 25 GO enrichment terms. Nodes represent GO terms, and edges represent shared genes between terms. Node size corresponds to gene count, edge thickness indicates the number of shared genes, and node color indicates the FDR

**Fig. 4:**
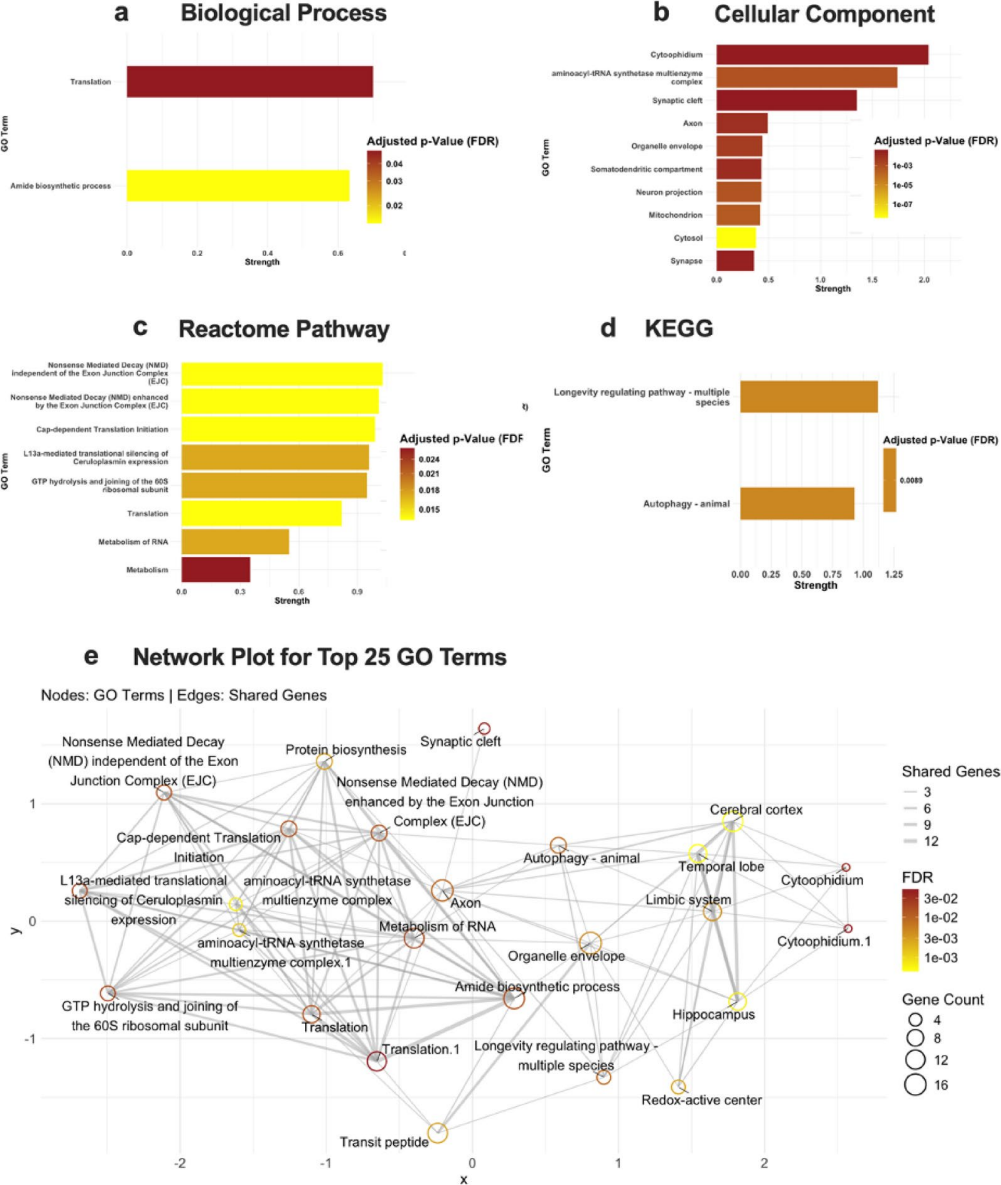
Female Gene Ontology (GO) with downregulated proteins: Enrichment Analysis for significantly downregulated proteins (p-value < 0.05) in female *Negr1*^*−/−*^ was performed using STRING analysis. GO term enrichment for (**a**) Biological Process, (**b**) Cellular Component, (**c**) Reactome Pathway, and (**d**) KEGG. Bar plots show the top enriched GO terms, with the x-axis representing enrichment strength, and bar color indicates the FDR (false discovery rate). (**e**) displays a network plot of the top 25 GO enrichment terms. Nodes represent GO terms, and edges represent shared genes between terms. Node size corresponds to gene count, edge thickness indicates the number of shared genes, and node color indicates the FDR

Functional enrichment of differentially abundant proteins was performed using STRING, as well as gene set enrichment analysis across GO categories (Biological Process, Molecular Function, and Cellular Component), and Reactome and KEGG pathways. The results, shown in Figs. 3 and 4, display enrichment strength on the x-axis and FDR (false discovery rate) indicated by bar color, for male (Fig. 3) and female downregulated proteins (Fig. 4), respectively. The list of genes for the corresponding GO term that are downregulated and upregulated for both males and females can be found in Supplementary Table S6-8.

Analysis revealed the most significantly downregulated biological processes in the male *Negr1*^*−/−*^ hippocampus included neurofilament bundle assembly, neurofilament cytoskeleton organization, postsynaptic cytoskeleton organization, calcium release, and myelination (Fig. 3a). These processes are primarily associated with the genes *Nefm*,* Nefl*,* Nefh*,* Ina*,* Arhgef7*,* Nol3*,* Pde4d*,* Slc8a1*,* Ank2*,* Akt1*,* Mbp*,* Mobp*,* Sirt2*,* Mag*,* and Qk.* The significantly downregulated molecular functions in the male *Negr1*^*−/−*^ hippocampus include structural components of the postsynaptic intermediate filament cytoskeleton, postsynaptic structural components, and synaptic structural components (Fig. 3b). The genes involved in these molecular functions are *Nefl*,* Ina*,* Dlg1*,* Nefh*,* and Rims1.* The genes *Nefm*,* Nefl*,* Ina*,* Nefh*,* Picalm*,* Mbp*,* Ermn*,* Llhl1*,* Dlg1*,* Sirt2n*,* Cnp*,* and Mag* are primarily associated with cellular components involved in Postsynaptic intermediate filament cytoskeleton, presynaptic intermediate filament cytoskeleton, Internode region of axon, myelin sheath, and neurofibrillary tangle are downregulated in male *Negr1*^*−/−*^ hippocampus (Fig. 3c). Significant downregulation of multiple genes associated with the Reactome pathway RHO GTPase cycle, including *Arhgef18*,* Samm50*,* Csk*,* Arhgdia*,* Golga2*,* Pkp4*,* Arhgef7*,* Rab7*,* Fam169a*,* Iqgap1*,* Arhgap12*,* Ktn1*,* Picalm*,* and Tjp2*, was identified in GO analysis from male *Negr1*^*−/−*^ hippocampus, affecting the neuronal cytoskeleton development, such as axon and dendrite growth (Fig. 3d).

Enrichment Analysis for significantly upregulated proteins in male *Negr1*^*−/−*^ hippocampus revealed mitochondrion organization as the most significantly upregulated biological process, and the genes involved are *Cxadr*,* Snx7*,* Tomm7*,* Map1lc3b*,* Bloc1s2*,* Dap3*,* Sco1*,* Tomm5*,* Ndufb2*,* Ndufaf2*, and *Atpaf1.* Mitochondria and the intracellular membrane-bound organelle are the key cellular components that are upregulated in male *Negr1*^*−/−*^ hippocampus. *Psmb4*,* Fdxr*,* Nt5c*,* Ppid*,* Tomm7*,* Map1lc3b*,* Isca1*,* Mrps21*,* Bloc1s2*,* Dap3*,* Sco1*,* Tomm5*,* Slc25a42*,* Gmppb*,* Hagh*,* Ndufb2*, and *Ndufaf2* are the genes that are upregulated and involved in mitochondrial function. The Reactome pathway, including cellular response to chemical stress, with the genes *​​Psmb4*,* Map1lc3b*,* Blvrb*,* Sco1*,* Skp1a*, and *Tbl1xr1* and PINK1-PRKN-mediated mitophagy with the genes *Tomm7*,* Map1lc3b*, and *Tomm5* were upregulated in male *Negr1*^*−/−*^ hippocampus, refer to Supplementary Figure S1 and Table S7.

In females, enrichment analysis showed results for biological process, cellular component, reactome pathway and KEGG. In females, the downregulated biological processes in *Negr1*^*−/−*^ hippocampus included translation and amide biosynthetic processes. The genes involved in these biological process are *Gcdh*,* Eif2b2*,* Rars*,* Etf1*,* Eif3a*,* Dars*,* Aimp2*,* Rpl22l1*,* Nanp*,* Rpl5*,* Rplp2*,* Rpl10*,* Acsl6*,* Mrps17*, and *Eif4a1* (Fig. 4a). Several cellular components are significantly downregulated in the female *Negr1*^*−/−*^ hippocampus. These included the cytophidium, associated with the genes *Ctps* and *Ctps2*, the aminoacyl-tRNA synthetase multienzyme complex, involving *Rars*,* Dars*,* Aimp2*, and *Rpl5*, and the synaptic cleft, linked to *Adgrb3*,* Agrn*, and *Nlgn1* (Fig. 4b). Genes that are downregulated in female *Negr1*^*−/−*^ hippocampus - *Prkacb*,* Ppp2ca*,* Rptor*,* Prkaa1*,* Nrbf2*,* Bcl2l1*,* Camkk2*,* Clpb*, and *Cryab* are involved in KEGG pathways such as Longevity regulating pathway - multiple species and autophagy (Fig. 4c). Reactome pathways, including Nonsense Mediated Decay (NMD) independent of the Exon Junction Complex (EJC), Nonsense Mediated Decay (NMD) enhanced by the Exon Junction Complex (EJC), and Cap-dependent Translation Initiation, are also downregulated in female *Negr1*^*−/−*^ hippocampus. *Etf1*,* Rpl22l1*,* Upf1*,* Rpl5*,* Rplp2*,* Rpl10*,* Ppp2ca*,* Eif2b2*,* Eif3a*, and *Eif4a1* are the genes involved in these reactome pathways (Fig. 4d). No significant enrichment was found in female upregulated genes in the STRING analysis due to a smaller number of DA in the female hippocampus.

A network plot of the top 25 GO enrichment terms that are downregulated in both male and female *Negr1*^*−/−*^ hippocampus was generated separately, in which nodes represent individual GO terms and edges represent shared genes between them (Figs. 3e and 4e). Node size is proportional to the number of genes associated with each GO term, while edge thickness reflects the extent of gene overlap. Node color indicates the FDR, allowing for the visualization of both functional relationships and the statistical significance of enrichment. This network highlights clusters of highly interconnected GO terms, suggesting shared biological pathways and functional coherence among the enriched terms. For the set of 66 upregulated proteins in male *Negr1*^*−/−*^ hippocampus, enrichment analysis did not yield several statistically significant pathway associations, likely due to the limited dataset size, which reduces statistical overlap with curated pathway annotations.

**Fig. 5:**
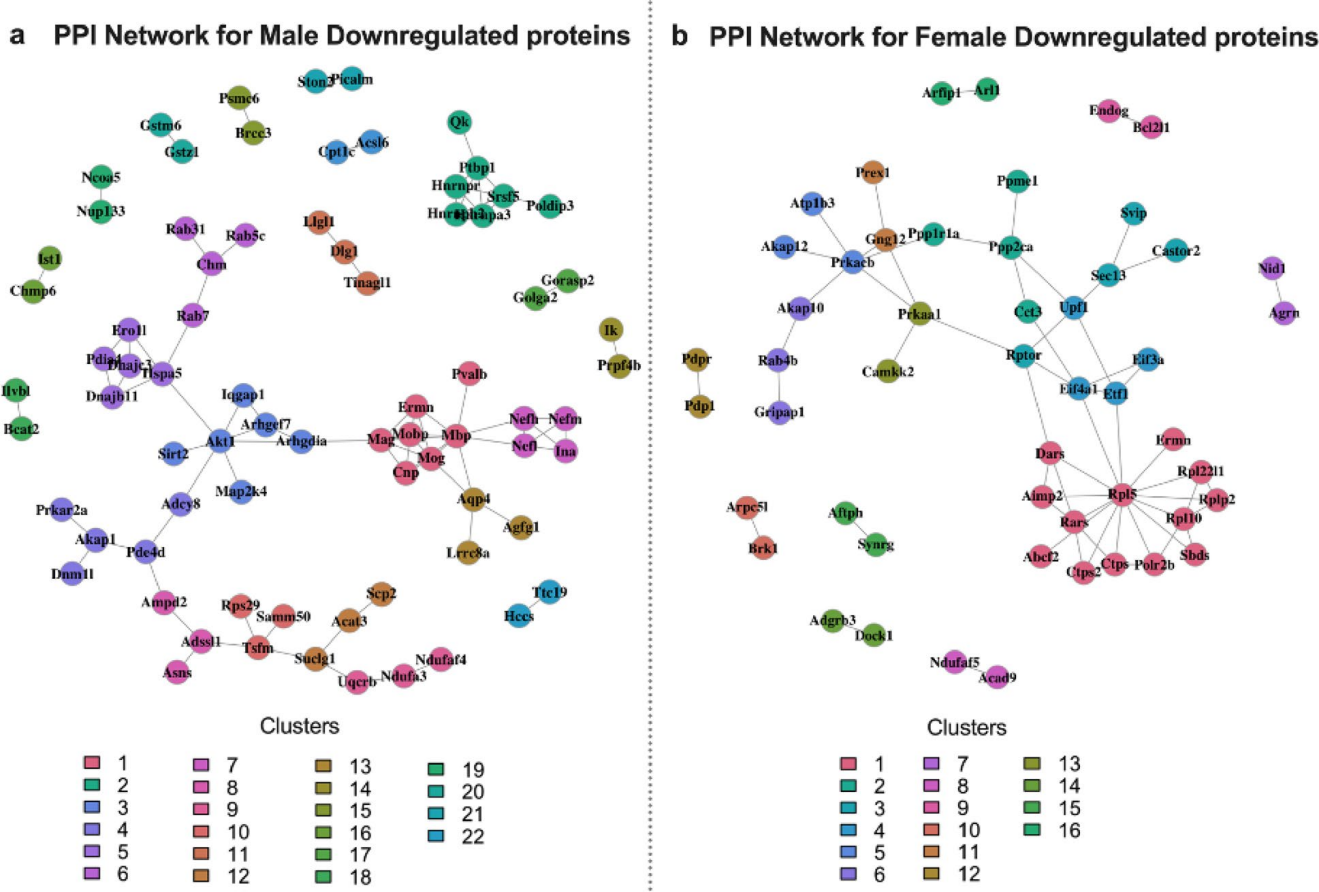
Protein-Protein Interaction (PPI) Network of male and female downregulated proteins clustered by Markov Clustering Algorithm (MCL). (**a**) The figure displays a high-confidence protein-protein interaction (PPI) network for male downregulated proteins. (**b**) The figure displays a high-confidence protein-protein interaction (PPI) network for female downregulated proteins. (**a**-**b**) The PPI network was constructed from STRING database interactions with high confidence (combined score > 0.6). Each node represents individual proteins, and edges represent physical or functional interactions. The network was clustered using the Markov Clustering Algorithm (MCL) with an inflation parameter 2.0 to identify functional modules. Each color indicates a distinct functional cluster

The proteins are grouped based on their biological functions. Figure 5a and 5b represent the PPI network for male and female downregulated proteins, respectively. The legend coding, along with descriptions for specific clusters, is provided in Supplementary Table S9-10. The key clusters that are downregulated in *Negr1*^*−/−*^ male hippocampus are oligodendrocyte development, neuron projection regulation, cAMP-dependent protein kinase activity, neurofilament cytoskeleton organization, and myelin sheath components.

In the male downregulated PPI network, a prominent cluster centered around *Pvalb* (Parvalbumin or PV) was identified, comprising key myelin sheath-associated proteins, including *Ermn*,* Mobp*,* Mbp*,* Mag*,* Mog*, and *Cnp*, as well as neurofilament cytoskeleton components such as *Nefh*,* Nefm*,* Nefl*, and *Ina*. This cluster points to a potential disruption in myelin sheath integrity and cytoskeletal organization. Pvalb was only connected to Mbp; Mbp actually seemed much more central because it was connected to both the myelin and the cytoskeleton proteins, plus the yellow cluster. In the female *Negr1*^*−/−*^ hippocampus, the key downregulated clusters are aminoacyl-tRNA synthetase multienzyme complex, serine/threonine phosphatase complex and calcineurin-NFAT signaling, amino acid regulation of mTORC1, cAMP-dependent protein kinase complex, laminin binding, and NADH dehydrogenase complex assembly.

### NEGR1–PV colocalization in the rat brain and human hippocampus

While the anatomical distribution of Negr1 in the central nervous system has been widely documented, the functional signals mediated by Negr1-expressing neurons remain poorly defined. In the male downregulated PPI network, Pvalb (Parvalbumin, PV) emerged around the central node, highlighting its potential link to *Negr1* deficiency. This prompted us to investigate the localization of Negr1 in relation to PV.

In our previous studies [15, 19], we examined Negr1 localization in Wt mouse brain tissue using immunohistochemistry. We also reported a reduced number of parvalbumin (PV)-positive interneurons in the hippocampus of 7-month-old male *Negr1*^*−/−*^ mice [13]. To determine whether these localization patterns are conserved across rodent species, we extended the analysis to rat brain tissue, which provides greater anatomical resolution due to its larger size. To further strengthen translational relevance, complementary analyses were performed in human brain tissue. We therefore examined Negr1–PV colocalization in rat and human hippocampus to explore this potential mechanistic connection using immunohistochemical analysis.

**Fig. 6:**
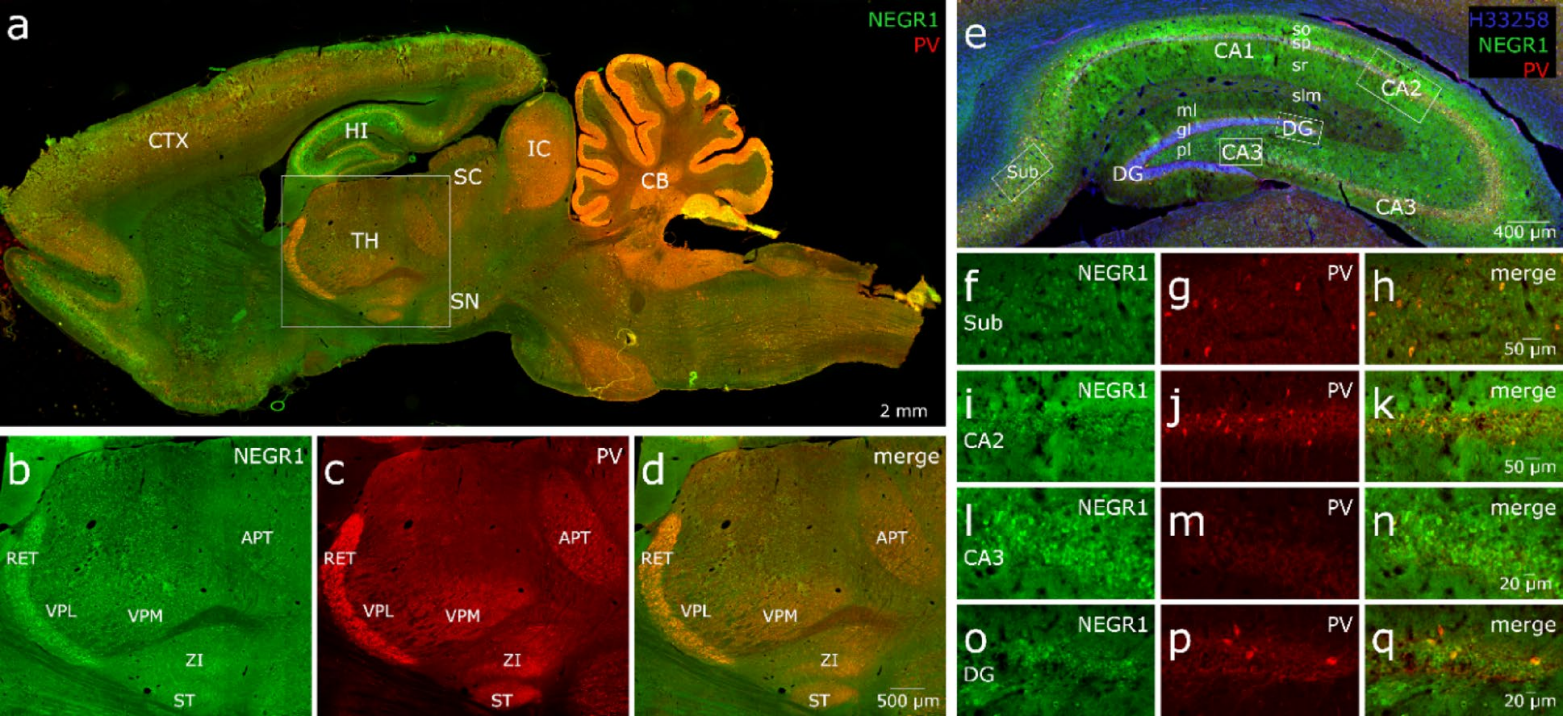
NEGR1–PV colocalization in the rat brain sagittal view (**a**) detected by immunostaining with primary antibody for NEGR1 in green, Parvalbumin (PV) in red. Panels **b**-**d** illustrate NEGR1–PV colocalization in distinct subregions of the thalamus. Panels **e**-**q** illustrate an overview of hippocampal subregions, subiculum (**f**–**h**), CA2 region of Ammon’s horn (**i**-**k**), CA3 region (**l**-**n**), and dentate gyrus (**o**-**q**). Abbreviations: cerebral cortex (CTX), hippocampus (HI), thalamus (TH), cerebellum (CB), substantia nigra (SN), superior colliculus (SC), inferior colliculus (IC), anterior pretectal nucleus (APT), reticular nucleus (RET), subthalamic nucleus (ST), ventral posterolateral nucleus (VPL), ventral posteromedial nucleus (VPM), zona incerta (ZI), dentate gyrus (DG), stratum oriens (so), stratum pyramidale-pyramidal layer (sp), stratum radiatum (sr), molecular layer of DG (ml), granule cell layer of DG (gl), polymorphic layer of DG (pl). Scale bars: (**a**) 2 mm, (**b**-**d**) 500 μm, (**e**) 400 μm (**f**-**k**) 50 μm and (**i**-**q**) 20 μm

**Fig. 7:**
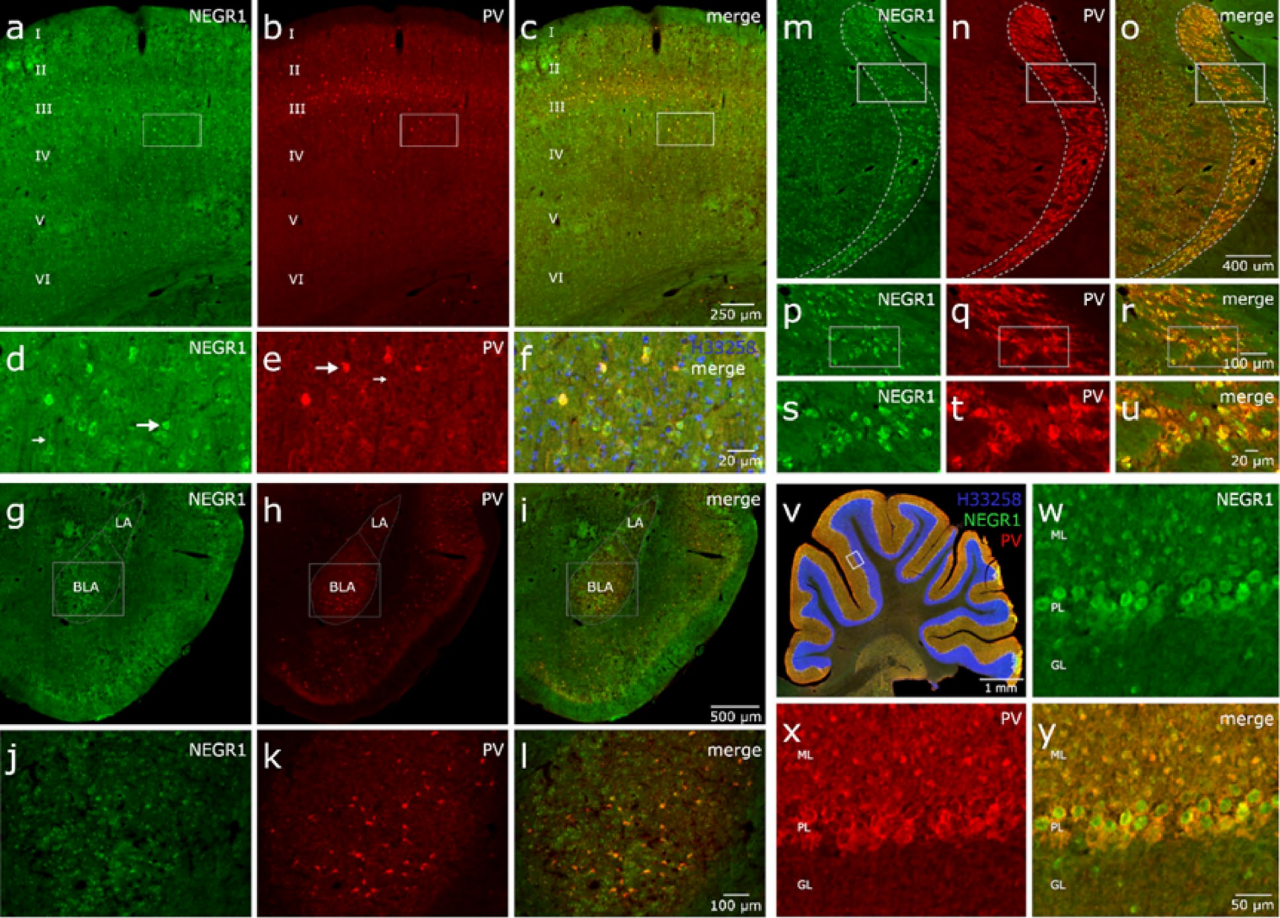
Images showing detailed analysis of Negr1 (green) and PV (red) nucleus (blue) immunostainings at different rat brain areas. NEGR1 and PV colocalization in the rat somatosensory cortex (**a**-**f**), amygdala (**g**-**l**), reticular nucleus (**m**-**u**) and cerebellum (**v**-**y**). Scale bars: (**a**-**c**) 250 μm, (**d**-**f**) 20 μm, (**g**-**i**) 500 μm, (**j**-**l**, **p**-**r**) 100 μm, (**m**-**o**) 400 μm, (**s**-**u**) 20 μm, (**v**) 1 mm, (**w**-**y**) 50 μm

In rat brain sections, NEGR1 immunoreactivity was predominantly observed in the neuronal somata, with lower levels detected in neuronal processes of hippocampus, cortex, thalamus, amygdala, substantia nigra, superior colliculus, inferior colliculus, and cerebellum Fig. 6, 7. A punctate labeling pattern was also apparent. PV immunoreactivity displayed a diffuse distribution, labeling both cell bodies and processes. Colocalization analysis demonstrated that the majority of PV+ inhibitory neurons also expressed NEGR1. In the cerebral cortex, both NEGR1 and PV were broadly expressed, except in layer I (molecular layer). NEGR1 expression was uniform across cortical regions and layers, whereas PV expression varied by region and layer. In all cortical areas, nearly all PV+ neurons also expressed NEGR1, while only a small proportion of NEGR1-positive cells were PV+ (Fig. 6a). In the somatosensory cortex, NEGR1 was evenly expressed across all layers (Fig. 7a-f). PV expression was strongest in layer III, with no PV-immunoreactive cells detected in layers V and VI. All PV+ cells in this region also expressed NEGR1.

In the thalamus (Fig. 6b-d), NEGR1 was robustly expressed throughout, with the most intense signal detected in the reticular nucleus. PV expression was also strongest in the reticular nucleus, where it colocalized extensively with NEGR1 (Fig. 7m-u). In addition, PV+ fibers and, to a lesser extent, small PV+ cell bodies were observed in other thalamic regions, some of which partially colocalized with NEGR1. Prominent PV labeling was also present in the ventral posteromedial (VPM) and ventral posterolateral (VPL) nuclei, as well as in the zona incerta (ZI) and the subthalamic nucleus (STN) (Fig. 6b-d). In all these areas, PV+ neurons expressed NEGR1, or NEGR1 + cells were surrounded by PV-positive processes.

In the hippocampus, as shown in Fig. 6e, NEGR1 was strongly expressed throughout the pyramidal cell layer of all Ammon’s horn fields (CA1–CA3) and in the granule cell layer of the dentate gyrus (DG). NEGR1 + were also observed in stratum oriens, stratum radiatum, and stratum lacunosum-moleculare. A high density of NEGR1 + neurons was present in the terminal region of CA3 bordering the DG (sometimes referred to as CA4), as well as in the molecular and polymorphic layers of the DG. PV displayed a largely similar distribution but differed from NEGR1 in that large, clearly defined neurons with extensive processes and a dense network of PV+ fibers were prominent in the pyramidal cell layer. Most PV+ cells in the hippocampus co-expressed NEGR1. Region-specific hippocampal analysis revealed distinct patterns (Fig. 6f–q). In the subiculum (Fig. 6f–h), numerous NEGR1 + and PV+ cells were present, with all PV+ neurons also expressing NEGR1. In CA2 (Fig. 6i–k), NEGR1 expression was strong in the pyramidal cell layer; while not all NEGR1 + cells were PV+, all PV+ cells co-expressed NEGR1. In CA3 (Fig. 6l–n), NEGR1 expression was high and accompanied by abundant PV-positive processes, though PV+ cell bodies were relatively sparse. In the DG (Fig. 6o–q), the granule cell layer exhibited widespread NEGR1 expression alongside PV+ fibers and PV+ neurons, all of which also expressed NEGR1. The amygdala images show that both NEGR1 and PV are strongly expressed, with substantial colocalization. Expression of both markers was particularly enriched in the basolateral amygdala (Fig. 7 g-l). NEGR1 and PV were both strongly expressed in the cerebellum and colocalized throughout the molecular layer and in Purkinje cells. A small number of NEGR1-positive cells were present in the granule cell layer, which also contained PV-positive fibers and, in rare cases, PV-positive cell bodies (Fig. 7v-y).

We also quantified NEGR1–PV colocalization in hippocampal regions; all PV+ cells were counted across the full extent of the hippocampus in both experimental animals (8 and 6 sections per animal). The proportion of PV-positive cells also expressing NEGR1 was calculated separately for the subiculum, combined Ammon’s horn fields (CA1–CA3), and the dentate gyrus. On average, 94% of PV+ cells in the subiculum, 96% in Ammon’s horn, and 88% in the dentate gyrus were also NEGR1+. Across the entire hippocampus (without regional subdivision), an average of 93% of PV+ cells expressed NEGR1. These values should be interpreted with caution, as the immunopositive signal for both proteins were not strictly binary but varied in intensity (high, medium, low, or subthreshold).

**Fig. 8:**
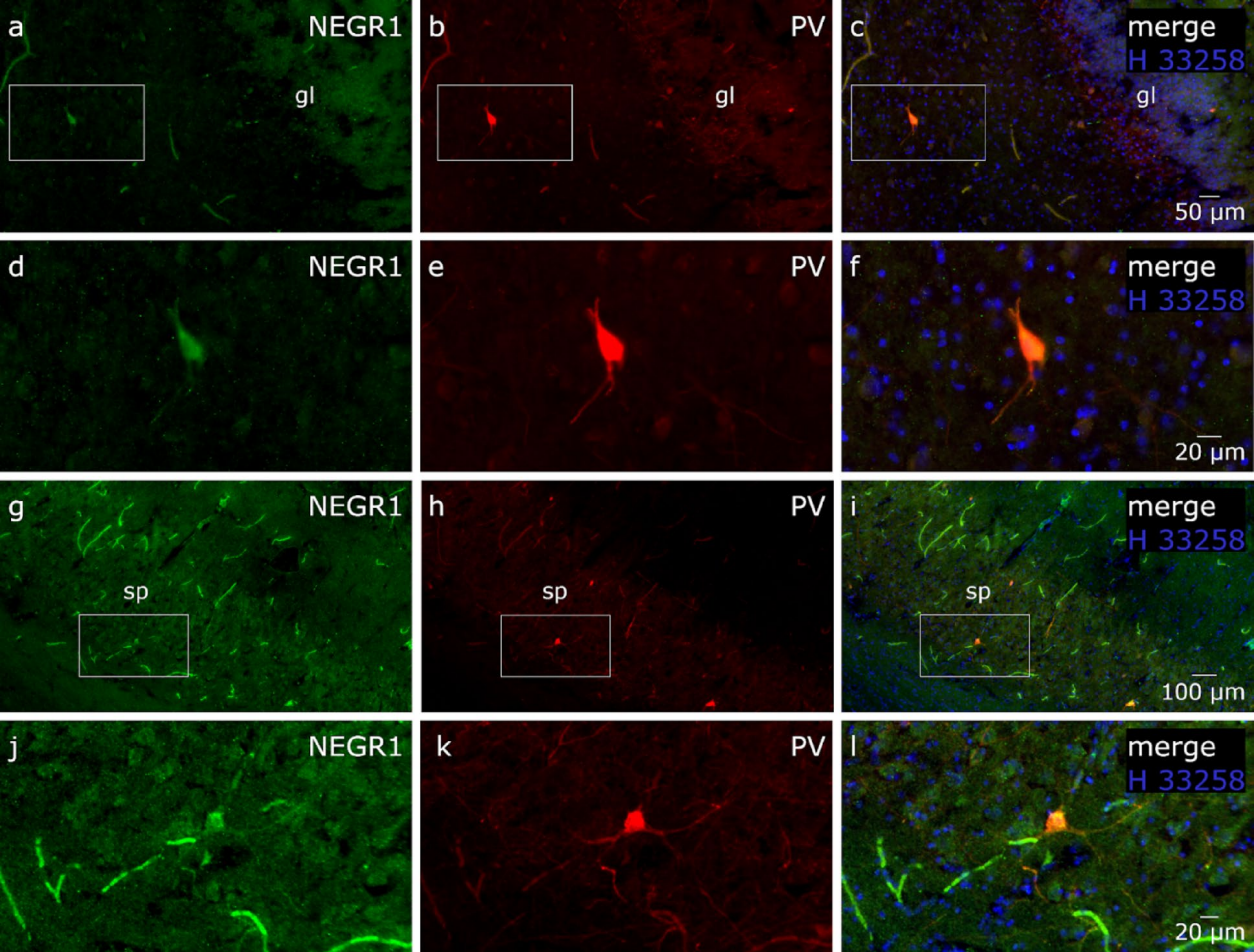
NEGR1 and PV colocalization in the human hippocampus. PV+ cells co-expressing NEGR1 are present in both the dentate gyrus (**a**–**f**) and the CA2 region (**g**–**l**) of the human hippocampus. Scale bar: (**a**-**c**) 50 μm, (**d**-**f**, **j**-**l**) 20 μm and (**j**-**i**) 100 μm

PV expressions in the human hippocampus follow a pattern similar to that observed in the rat brain. In addition to individual, clearly defined PV+ neurons with long processes across hippocampal regions. Weakly labeled NEGR1 + cells can be distinguished in the pyramidal layers of CA1–CA3, with low immunoreactivity also observed in the granule layer of the dentate gyrus. Importantly, all PV+ neurons co-express NEGR1. NEGR1 and PV colocalization in human hippocampal cells is illustrated in Fig. 8.

## Discussion

In this study, we employed an untargeted quantitative proteomics approach to obtain large-scale baseline comparisons of protein abundance changes in the hippocampus of *Negr1*^*−/−*^ mice with their Wt littermates in both male and female mice. Male and female mice exhibited divergent proteomic profiles and underlying molecular mechanisms. By characterizing these sex-dependent proteomic differences in the *Negr1*^*−/−*^ hippocampus, this work provides a critical reference for the neuroscience community. We validated the translational relevance of NEGR1 by performing immunostaining to examine its cellular localization in rat and human postmortem brain samples. These findings provide a framework for future experimental studies.

The PCA plot, supported by PERMANOVA analysis, indicates a robust and significant separation between male and female samples. The F2 background (129S5/SvEvBrd × C57BL/6 N) of our *Negr1*^*−/−*^ mice incorporates substantial genetic diversity, resulting in increased individual variability in behavioral traits relevant to neurodevelopmental and psychiatric disorders [13, 45]. While this variability may reduce the separation of experimental groups in genotype unsupervised analyses, such as PCA plots for genotype separation, it reflects a more representative model of natural variation, enhances translational relevance, and does not compromise the reproducibility of our findings. Indeed, our previous studies with this mouse line have consistently demonstrated replicable behavioral results. Overall protein abundance in the hippocampus was higher in male mice than in females, likely reflecting region-specific sex differences in hippocampal subfield volumes, which are generally larger in males [46]. A total of 223 (56.5%) and 172 (41.3%) protein entries showed significantly regulated abundance in males and females, respectively. Our analysis found only 9 DA proteins that are commonly present in both male and female and are involved in fundamental cellular processes like amino acid metabolism, pH regulation, myelination, protein folding, intracellular trafficking, and cell signaling, highlighting their essential role in maintaining neuronal and glial homeostasis across sexes (details can be found in Supplementary Material 2, Table S1.2).

STRING PPI network analysis revealed sex-specific patterns, showing distinct functional clusters among the downregulated proteins. In males, *Negr1* deficiency resulted in downregulation of neurofilament subunits, as well as several myelin-associated proteins (*Nefm*,* Nefl*,* Nefh*,* Ina*,* Arhgef7*,* Nol3*,* Pde4d*,* Slc8a1*,* Ank2*,* Akt1*,* Mbp*,* Mobp*,* Sirt2*,* Mag*, and *Qk*). These proteins are fundamental for maintaining axonal scaffolding, oligodendrocyte-mediated conduction support. Oligodendrocytes not only insulate axons but also supply essential metabolic substrates to fast-spiking interneurons, and their dysfunction can disrupt signal timing and axonal integrity. The affected myelin proteins—*Mbp*, *Mag*, and *Cnp*—play core roles in myelin sheath formation and axon–glia domain organization, whereas *Mobp* and *Ermn* contribute to myelin stabilization during maturation [47]. The reduction of *Sirt2*, a deacetylase that regulates oligodendrocyte differentiation and microtubule dynamics, and *Sirt2* dysregulation can disrupt axonal transport and neuronal connectivity [48]. Moreover, downregulation of Rho GTPase cycle components further targets the actin cytoskeleton, thereby producing distinct effects on synaptic morphology, stability, functional plasticity, and has been associated with psychiatric diseases such as schizophrenia and depression, and also with neurodevelopmental disorders including autism spectrum disorders [49]. Interestingly, another recent study using *Negr1* overexpressing mice showed that similar myelination-regulating and Rho GTPases function genes, *Mbp*,* Mag* and *Golga2*, were downregulated in the mice displaying depression- and anxiety-like phenotypes, consistent with our findings and underscoring the relevance of our results [50]. *Negr1* has also been identified as a mediator of permissive axon–myelin interactions [51].

PPI network analysis revealed Pvalb, PV-centered cluster enriched in myelin-associated proteins, suggesting that *Negr1* deficiency disrupts an interneuron–oligodendrocyte axis essential for maintaining inhibitory synchrony. PV interneurons play a key role in orchestrating hippocampal oscillations and cognitive processing, and their dysfunction represents a hallmark of several major psychiatric and neurodegenerative disorders [52, 53]. Together, these findings suggest that *Negr1* deficiency may compromise cytoskeletal and myelin maintenance, leading to interneuron vulnerability and impaired network synchrony, which has been linked to the molecular mechanisms of several neuropsychiatric disorders like autism spectrum, schizo-affective disorders, depression, Alzheimer’s etc [54–57].

Conversely, males upregulated protein targets belong to mitochondrial organization and mitophagy-related proteins, suggesting activation of compensatory metabolic responses to maintain hippocampal neural circuitry under structural stress. Mitochondria in PV interneurons are strategically positioned to meet local energy and calcium requirements, and disruptions in their dynamics lead to compromised gamma oscillations and impaired behavioral regulation [54]. The observed enrichment pathways related to cellular stress response and PINK1–PRKN-mediated mitophagy suggest that *Negr1* deficiency in males may disrupt mitochondrial homeostasis, triggering adaptive turnover mechanisms to protect against oxidative and metabolic imbalances [58]. Importantly, a study showed that PV deficiency causes mitochondrial enlargement, likely as a compensatory response to increased energy demands [59]. Together, these findings provide intermutual mechanistic insights into the interneuron–oligodendrocyte axis, not only at the structural level but also highlighting PV vulnerability in relation to mitochondrial impairments that are only pronounced in males.

In females, downregulated proteins include several key proteins involved in translational control, synaptic organization, neuronal excitability, and intracellular trafficking (*Zwint*,* Armh3*,* Rap1gds1*,* Arfip1*,* Nrbf2*,* Rplp2*,* Rpl10*,* Rgs14*,* Aftph*,* Kcna4*,* Folh1*,* and Trappc10*). Enrichment analyses revealed downregulation of biological processes associated with translation and amide biosynthesis, as well as suppression of key components of the RNA surveillance and autophagy pathways. These coordinated reductions in ribosomal and translation initiation factors (*Eif2b2*,* Eif3a*,* Eif4a1*,* Rpl5*,* Rpl10*,* Rpl22l1*,* Rplp2*) impair the synthesis of proteins necessary for synaptic plasticity and memory formation, potentially compromising hippocampal function [60]. Interestingly, it is noted that the *eukaryotic initiation factor (eIF*) family plays a crucial role in female reproduction by regulating translation initiation, a process essential for the synthesis of proteins required for key events such as oogenesis, embryonic development, and implantation [61, 62]. Downregulation of proteins associated with Nonsense-Mediated Decay (NMD) and cap-dependent translation initiation further suggests compromised mRNA surveillance, potentially leading to the accumulation of abnormal RNA and proteostasis imbalance [63]. Additionally, the suppression of autophagy-related and longevity-regulating pathways implies impaired clearance of damaged proteins and organelles, a mechanism crucial for maintaining metabolic homeostasis and neuronal integrity under cellular stress [64]. This molecular signature aligns with reports that female brains often exhibit heightened sensitivity to disruptions in proteostatic and metabolic pathways, possibly reflecting sex-dependent differences in energy utilization and neuroprotective signaling [65, 66]. Recent attention has also focused on mRNA translation mechanisms—particularly those governed by mTORC1, ISR/eIF2, and eEF2/eEF2K pathways—which play critical roles in prenatal brain development, synaptic plasticity, and circuit remodeling. Dysregulation of these translational control pathways has been increasingly recognized as a hallmark of multiple neuropsychiatric and neurodevelopmental conditions [67].

Negr1 is expressed in both neuronal and glial cells in the mouse brain [68], including neurons and oligodendrocytes where it regulates neurite outgrowth and synapse formation and is upregulated in astrocytes during axonal regeneration, collectively contributing to neurodevelopmental processes such as neuronal migration, dendritic spine formation, synaptic connectivity, and structural plasticity [12, 28, 69]. Notably, *Negr1*^*−/−*^ mice show reduced expression of glial lipocalin-2 (Lcn2), an inflammatory mediator produced by astrocytes and microglia that influences adult hippocampal neurogenesis [14]. These findings suggest that NEGR1 may modulate neuronal–glial interactions and inflammatory pathways that contribute to altered hippocampal circuitry.

This is the first proteomics analysis of male and female *Negr1*^*−/−*^ mice, point to clear sex differences in hippocampal tissue. *Negr1* deficiency in the female hippocampus resulted in impaired protein synthesis and turnover, potentially weakening synaptic stability and neuronal resilience. In contrast, male hippocampi exhibited alterations in cytoskeletal organization, myelination, and mitochondrial function. This likely reflects inherent sex differences in hippocampal signaling and hormone-sensitive plasticity programs that modulate mTOR and ISR pathways [33, 70]. It is reasonable to question whether the observed sex differences in protein abundance in this study might stem from variations in the relative proportions of neuronal versus non-neuronal cell types. Sex differences in the hippocampal proteome may also reflect variations in astrocyte and microglia numbers between males and females, in addition to their metabolic differences [71]. Further studies based on these targets are warranted to elucidate the precise mechanistic role of these pathways and to assess their potential as therapeutic targets.

Our previous publication demonstrated a reduction of PV⁺ cells in male mice of *Negr1*^*−/−*^, providing initial validation of NEGR1’s association with inhibitory hippocampal circuitry [13]. To further assess translational relevance and cellular localization, we examined PV and NEGR1 in rat brain and human hippocampal sections. This was prompted by our male hippocampal proteomic analysis, which identified PV/Pvalb around the central node within a downregulated PPI cluster. Our unpublished data from age-matched (7-month-old) female mice did not show a reduction in PV-positive cells in the hippocampus. Therefore, we proceeded with further validation only in male mice, while considering the female findings as exploratory. Future studies are needed to investigate the mechanisms identified in the female hippocampus.

We demonstrate extensive colocalization of NEGR1 with PV interneurons across several brain regions in the rat, and within the hippocampus in humans, indicating a conserved role for NEGR1 in inhibitory circuit regulation across species. In rats, the widespread overlap of NEGR1 with PV-positive interneurons suggests that NEGR1 is expressed in key inhibitory neuronal populations, potentially modulating their connectivity and network activity to support behavioral and cognitive processing [50, 51]. Importantly, while most PV interneurons expressed NEGR1, not all NEGR1-positive cells were PV-positive, which also indicates that NEGR1 is not confined to inhibitory neurons and may also contribute to other regulatory processes. Notably, our recent study [72] independently demonstrated reduced hippocampal Pvalb mRNA expression in *Negr1*^*−/−*^ mice, with a significant genotype effect in males and a milder effect in females, particularly under social isolation stress. The convergence of proteomic and transcriptional findings across independent cohorts therefore supports the notion that Negr1 deficiency affects PV interneuron-related pathways in the hippocampus. The stronger effect observed in males in both datasets further suggests a potential sex-dependent vulnerability of inhibitory circuitry associated with Negr1 deficiency. Future studies will be required to determine whether these molecular changes translate into functional alterations of PV interneuron activity and hippocampal network dynamics [73].

Altogether, our findings indicated that several significantly altered proteins in *Negr1*^*−/−*^ mice have been previously linked to molecular mechanisms implicated in neuropsychiatric disorders, including autism spectrum disorder, schizo-affective disorders, major depression, and Alzheimer’s disease. Notably, proteins associated with myelination, synaptic regulation, and cellular signaling pathways show convergence with molecular signatures reported in these conditions. Although such overlaps do not establish a direct etiological relationship, they highlight shared biological pathways that may contribute to circuit dysfunction. Together with the behavioral phenotypes observed in *Negr1*^*−/−*^ mice, these molecular parallels support the model’s utility for investigating mechanisms relevant to neuropsychiatric disorders. Future studies will investigate sex-specific regulation of the identified proteins and their interaction with NEGR1 to better understand how these mechanisms contribute to the observed molecular and behavioral alterations, particularly in light of growing evidence that neuropsychiatric disorders display sex-dependent differences in prevalence and underlying biology.

The main limitation of this work is that the findings for females remain exploratory and require further confirmation. Estrous staging and hormonal levels were not assessed in this study, which we acknowledge as a limitation. We provided baseline sex-based observations under normal housing conditions, and future studies will include assessment of endocrine rhythms. Translational analyses in rat and human tissue may not fully capture dynamic in vivo changes, and causal mechanisms linking NEGR1 to PV interneuron function, myelination, or mitochondrial adaptations remain to be established. Further investigation into the mechanistic interactions between PV and NEGR1 will be crucial to determine how NEGR1 influences the stability and function of inhibitory circuits, including its potential influence on myelination and mitochondrial compensation in male mice. Future studies should prioritize validating female-specific target pathways, including RNA stability, longevity, and mitochondrial adaptations, to fully understand the sex-dependent roles of NEGR1 in the brain.

In conclusion, this first proteomic analysis of the male and female *Negr1*^*−/−*^ hippocampus provides direct evidence of widespread dysregulation of numerous proteins in both sexes compared to controls. Bioinformatic analyses of the differentially abundant proteins revealed sex-specific alterations in distinct pathways and protein interaction networks. Collectively, these molecular changes indicate a proteostasis-centered adaptive response in the female *Negr1*^*−/−*^ hippocampus, where translational regulation, RNA surveillance, and stress-response mechanisms are prioritized over structural remodeling. In contrast, the male hippocampus exhibits pronounced disruptions in cytoskeletal organization, myelination, mitochondrial function, and parvalbumin interneuron-related pathways, suggesting that *Negr1* deficiency directly impacts inhibitory circuit stability and energy metabolism. Colocalization analysis further revealed that *Negr1* is expressed within PV interneurons, suggesting a potential cell-autonomous role in inhibitory circuit maturation and highlighting cell-type-specific functions of NEGR1.

Together, these findings underscore sex-dependent mechanisms of hippocampal circuit vulnerability associated with *Negr1* deficiency and point to Negr1 as a key molecular node linking structural, metabolic, and inhibitory network alterations relevant to neuropsychiatric disease–related behavioral phenotypes. Therefore, our study highlights the novelty of sex-specific proteomic signatures, their translational relevance, and insights into network biology. 

## Supplementary Information

Below is the link to the electronic supplementary material.

Supplementary Material 1

Supplementary Material 2

Supplementary Material 3

Supplementary Material 4

## Data Availability

The datasets generated and/or analyzed during the current study are available from the corresponding authors on request.
