# Supplementary material for: Sex-specific changes in the hippocampal proteome of Negr1−/− mice: insight into the mechanisms of neuropsychiatric disorders
Source: Biol Sex Differ. 2026 Mar 26;17:96. doi: 10.1186/s13293-026-00890-0 (PMC13141394; doi:10.1186/s13293-026-00890-0)
Supplement: Supplementary file 4 — Supplementary Material 4 [file 13293_2026_890_MOESM4_ESM.docx]

**Supplementary Material 1**

**Sex-specific changes in the hippocampal proteome of *Negr1^-/-^* mice: insight into the mechanisms of neuropsychiatric disorders**

Srirathi Muthuraman^1^, Mohan Jayaram^1^, Liisi Promet^1^, Toomas Jagomäe^1^, Arun Kumar Devarajan^2^, Andreas-Christian Hade^3^, Mari-Anne Philips^1^, Katyayani Singh^1*^, Eero Vasar^1^

**Supplementary Table S1**: **Demographic and postmortem characteristics of human brain samples used for immunohistochemical analysis.** Study subjects from whom perfused human brain tissues were collected at the Department of Pathological Anatomy and Forensic Medicine, University of Tartu, Estonia, and the Estonian Forensic Science Institute, Tallinn, Estonia, and subsequently processed for immunohistochemical analyses. PMI - post mortem interval; M-Male; F- Female; warm time - time before cold storage at 4 °C.

| ID | Age | Sex | Brain pH | PMI (hours) | Warm time (hours) | Cause of death |
| --- | --- | --- | --- | --- | --- | --- |
| P1 | 42 | M | 6,37 | 18 | 1 | Drug poisoning |
| P2 | 27 | M | 6,44 | 3 | 1 | Drug poisoning |
| P3 | 28 | F | 6,58 | 43 | 1.5 | Drug poisoning |
| P4 | 37 | M | 6,80 | 14 | 5 | Chronic alcohol-induced multi-organ failure |
| P5 | 21 | F | 6,39 | 44 | 1.5 | Sudden cardiac death |

**Supplementary Table S1.1:** Top 12 Upregulated proteins in male and female *Negr1^-/-^* hippocampus with their Gene, UniProt ID, Protein Name, P-value (pval), and differential abundance (Regulation).

| **Gene** | **UniProt ID** | **Protein Name** | **pval** | **Regulation** |
| --- | --- | --- | --- | --- |
| Isca1 | Q9D924 | Iron-sulfur cluster assembly 1 homolog, mitochondrial | 0.0017 | Upregulated in males |
| Fdxr | Q61578 | NADPH:adrenodoxin oxidoreductase, mitochondrial | 0.00219 | Upregulated in males |
| Skp1 | Q9WTX5 | S-phase kinase-associated protein 1 | 0.0029 | Upregulated in males |
| Scrg1 | O88745 | Scrapie-responsive protein 1 | 0.0049 | Upregulated in males |
| Rtn4rl2 | Q7M6Z0 | Reticulon-4 receptor-like 2 | 0.0052 | Upregulated in males |
| Ddx23 | D3Z0M9 | DEAD-Box Helicase 23 | 0.0055 | Upregulated in males |
| Cxadr | P97792 | Coxsackievirus and adenovirus receptor homolog | 0.0070 | Upregulated in males |
| Ppid | Q9CR16 | Peptidyl-prolyl cis-trans isomerase D | 0.0076 | Upregulated in males |
| Ankrd17 | Q99NH0 | Ankyrin repeat domain-containing protein 17 | 0.0092 | Upregulated in males |
| Hagh | Q99KB8 | Hydroxyacylglutathione hydrolase, mitochondrial | 0.0105 | Upregulated in males |
| Map1lc3b | Q9CQV6 | Microtubule-associated proteins 1A/1B light chain 3B | 0.0123 | Upregulated in males |
| Tomm5 | B1AXP6 | Mitochondrial import receptor subunit TOM5 homolog | 0.0128 | Upregulated in males |
| Hccs | P53702 | Cytochrome c-type heme lyase | 0.0002 | Downregulated in males |
| Top2b | Q64511 | DNA topoisomerase 2-beta | 0.0008 | Downregulated in males |
| Arhgef18 | Q6P9R4 | Rho guanine nucleotide exchange factor 18 | 0.001 | Downregulated in males |
| Agfg1 | Q8K2K6 | Arf-GAP domain and FG repeat-containing protein 1 | 0.0018 | Downregulated in males |
| Ergic1 | Q9DC16 | Endoplasmic reticulum-Golgi intermediate compartment protein 1 | 0.0023 | Downregulated in males |
| Ilvbl | Q8BU33 | Acetolactate synthase-like protein | 0.0023 | Downregulated in males |
| Smarcal1 | Q8BJL0 | SWI/SNF-related matrix-associated actin-dependent regulator of chromatin subfamily A-like protein 1 | 0.0028 | Downregulated in males |
| Myo1d | Q5SYD0 | Unconventional myosin-Id | 0.004 | Downregulated in males |
| Asns | Q61024 | Asparagine synthetase [glutamine-hydrolyzing] | 0.0041 | Downregulated in males |
| Pvalb | Q545M7 | Parvalbumin alpha | 0.0041 | Downregulated in males |
| Ncoa5 | Q91W39 | Nuclear receptor coactivator 5 | 0.0051 | Downregulated in males |
| Evi5l | Q3U1G0 | Ecotropic Viral Integration Site 5 Like | 0.0053 | Downregulated in males |
| Parl | Q5XJY4 | Presenilins-associated rhomboid-like protein, mitochondrial;P-beta | 0.0007 | Upregulated in females |
| Samhd1 | Q60710 | Deoxynucleoside triphosphate triphosphohydrolase SAMHD1 | 0.0023 | Upregulated in females |
| Bcar1 | Q61140 | Breast cancer anti-estrogen resistance protein 1 | 0.0064 | Upregulated in females |
| Uqcr11 | Q9CPX8 | Cytochrome b-c1 complex subunit 10 | 0.0066 | Upregulated in females |
| Brinp2 | Q6DFY8 | BMP/retinoic acid-inducible neural-specific protein 2 | 0.0073 | Upregulated in females |
| Mmgt1 | Q8K273 | Membrane magnesium transporter 1 | 0.0092 | Upregulated in females |
| Lsm12 | Q9D0R8 | Protein LSM12 homolog | 0.01 | Upregulated in females |
| Ppm1k | Q8BXN7 | Protein phosphatase 1K, mitochondrial | 0.016 | Upregulated in females |
| Clptm1 | Q8VBZ3 | Cleft lip and palate transmembrane protein 1 homolog | 0.0168 | Upregulated in females |
| Snx32 | Q80ZJ7 | Sorting nexin-32 | 0.018 | Upregulated in females |
| Golph3 | Q9CRA5 | Golgi phosphoprotein 3 | 0.0182 | Upregulated in females |
| Nedd4 | P46935 | E3 ubiquitin-protein ligase NEDD4 | 0.0183 | Upregulated in females |
| Zwint | Q9CQU5 | ZW10 interactor | 0.0013 | Downregulated in females |
| Armh3 | Q6PD19 | Armadillo Like Helical Domain Containing 3 | 0.002 | Downregulated in females |
| Rap1gds1 | E9Q912 | Rap1 GTPase-GDP Dissociation Stimulator 1 | 0.0023 | Downregulated in females |
| Arfip1 | G5E8V9 | Arfaptin-1 | 0.0026 | Downregulated in females |
| Nrbf2 | Q8VCQ3 | Nuclear receptor-binding factor 2 | 0.0037 | Downregulated in females |
| Rplp2 | P99027 | 60S acidic ribosomal protein P2 | 0.004 | Downregulated in females |
| Rpl10 | Q6ZWV3 | Ribosomal protein;60S ribosomal protein L10a | 0.0047 | Downregulated in females |
| Rgs14 | P97492 | Regulator of G-protein signaling 14 | 0.0056 | Downregulated in females |
| Aftph | Q80WT5 | Aftiphilin | 0.0061 | Downregulated in females |
| Kcna4 | Q61423 | Potassium voltage-gated channel subfamily A member 4 | 0.0066 | Downregulated in females |
| Folh1 | O35409 | Glutamate carboxypeptidase 2 | 0.0072 | Downregulated in females |
| Trappc10 |  | Trafficking protein particle complex subunit 10 | 0.009 | Downregulated in females |

**Supplementary Table S1.2:** Common differentially abundant proteins in both male and female hippocampus with their protein codes (Protein), Protein name, Differential abundance, and Neurological relevance with citation (references below the table)

| **Protein** | **Protein name** | **Differential abundance** | **Neurological relevance** | **Citations** |
| --- | --- | --- | --- | --- |
| Bcat2 | Branched-chain-amino-acid aminotransferase, mitochondrial | Downregulated in both male and female hippocampus | Supports neurotransmitter balance and central nervous system function. | Choi *et al.,* 2024 |
| Ca2;Car2 | Carbonic anhydrase 2 | Downregulated in both male and female hippocampus | Involved in neurodevelopment and depletion of Ca2 is associated with the pathogenesis of cerebral calcifications and mental retardation. | Kida *et al.,* 2006 |
| Ermn | Ermin | Downregulated in both male and female hippocampus | Marker of myelinating oligodendroglia and also plays a role in cytoskeletal rearrangements. | Brockschnieder *et al.,* 2006 |
| Ero1l | ERO1-like protein alpha | Downregulated in both male and female hippocampus | Involved in the endoplasmic reticulum stress response and contributes to the neuroprotective mechanism. | Jin *et al.,* 2025 |
| Fam45a | Fam45a | Downregulated in both male and female hippocampus | Plays a crucial role in neurite outgrowth. | Li *et al.,* 2025 |
| Lsm12 | Protein LSM12 homolog | Downregulated in male hippocampus and upregulated in female hippocampus | Functions in neuroprotective pathways sustaining nucleocytoplasmic transport. | Lee *et al.,* 2020 |
| Nanp | N-acylneuraminate-9-phosphatase | Downregulated in both male and female hippocampus |  |  |
| Sf1 | Splicing factor 1 | Downregulated in both male and female hippocampus | Involved in the neurodevelopmental disorder. | Bou-Rouphael *et al.,* 2025 |
| Snx32 | Sorting nexin-32 | Upregulated in both male and female hippocampus | Regulates cargo sorting and neurite outgrowth. | Sugatha *et al.,* 2023 |

**References:**

1. Choi BH, Hyun S, Koo SH. The role of BCAA metabolism in metabolic health and disease. Experimental & Molecular Medicine. 2024 Jul 1;56(7):1552–9. <https://doi.org/10.1038/s12276-024-01263-6>
2. Kida E, Palminiello S, Wisniewski KE, Walus M, Albertini G, Wierzba-Bobrowicz T, et al. Carbonic Anhydrase II in the Developing and Adult Human Brain. Journal of Neuropathology and Experimental Neurology. 2006 Jul 1;65(7):664–74. <https://doi.org/10.1097/01.jnen.0000225905.52002.3e>
3. Brockschnieder D, Riethmacher D, Sabanay H, Peles E. Ermin, A Myelinating Oligodendrocyte-Specific Protein That Regulates Cell Morphology. The Journal of Neuroscience. 2006 Jan 18;26(3):757–62. <https://doi.org/10.1523/jneurosci.4317-05.2006>
4. Jin H, Chen L, Huan Y, Chu G, Zhao H, Gong S, et al. Functional characterization of key protein biomarkers in spontaneous intracerebral hemorrhage pathogenesis: Structure and function of NUAK1 protein and ERO1L protein macromolecules. International journal of biological macromolecules. 2025 May 1;309(Pt 2):142959. <https://doi.org/10.1016/j.ijbiomac.2025.142959>
5. Li A, Zhang J, Ma C, Qi L, Hu Q, Li Q, et al. Endosomal protein DENND10 promotes developmental competence of neurite extension. iScience. 2025 May 1;28(5):112385. <https://doi.org/10.1016/j.isci.2025.112385>
6. Lee J, Kim YK, Yoon KJ, Park TE, Lim C, Lee G, et al. LSM12-EPAC1 defines a neuroprotective pathway that sustains the nucleocytoplasmic RAN gradient. PLOS Biology. 2020 Dec 23;18(12):e3001002. <https://doi.org/10.1371/journal.pbio.3001002>
7. Bou-Rouphael J, Cospain A, Courtin T, Keren B, Marie C, Lesieur-Sebellin M, et al. Heterozygous pathogenic variants in the splicing factor SF1 lead to a large spectrum of neurodevelopmental disorders. American journal of human genetics. 2025 Nov 1;112(11):2605–24. <https://doi.org/10.1016/j.ajhg.2025.09.001>
8. Sugatha J, Jose A, Raj P, Datta S, Swaminathan U, Jaimon E, et al. Insights into cargo sorting by SNX32 and its role in neurite outgrowth. eLife. 2023 May 9;12. <https://doi.org/10.7554/elife.84396>


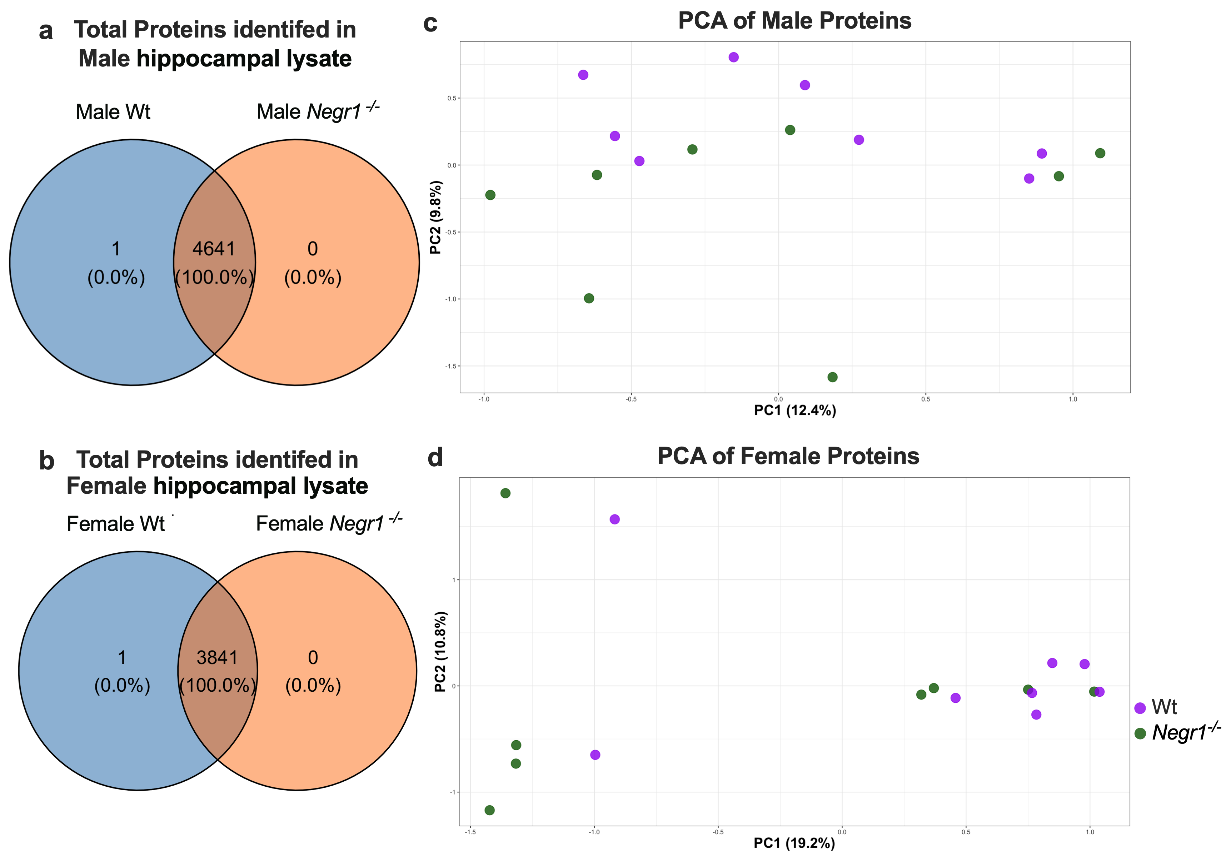


**Supplementary Figure 1:** (a) Venn diagram of the total number of proteins identified in male Wt and *Negr1 ^-/-^* hippocampal lysate, showing the unique and common proteins. (b) Venn diagram of the total number of proteins identified in male Wt and *Negr1 ^-/-^* hippocampal lysate, showing the unique and common proteins. (c) PCA plot of male hippocampal protein expression profiles. (d) PCA plot of female hippocampal protein expression profiles. Each point represents an individual mouse (purple: Wt; green: *Negr1 ^-/-^*).


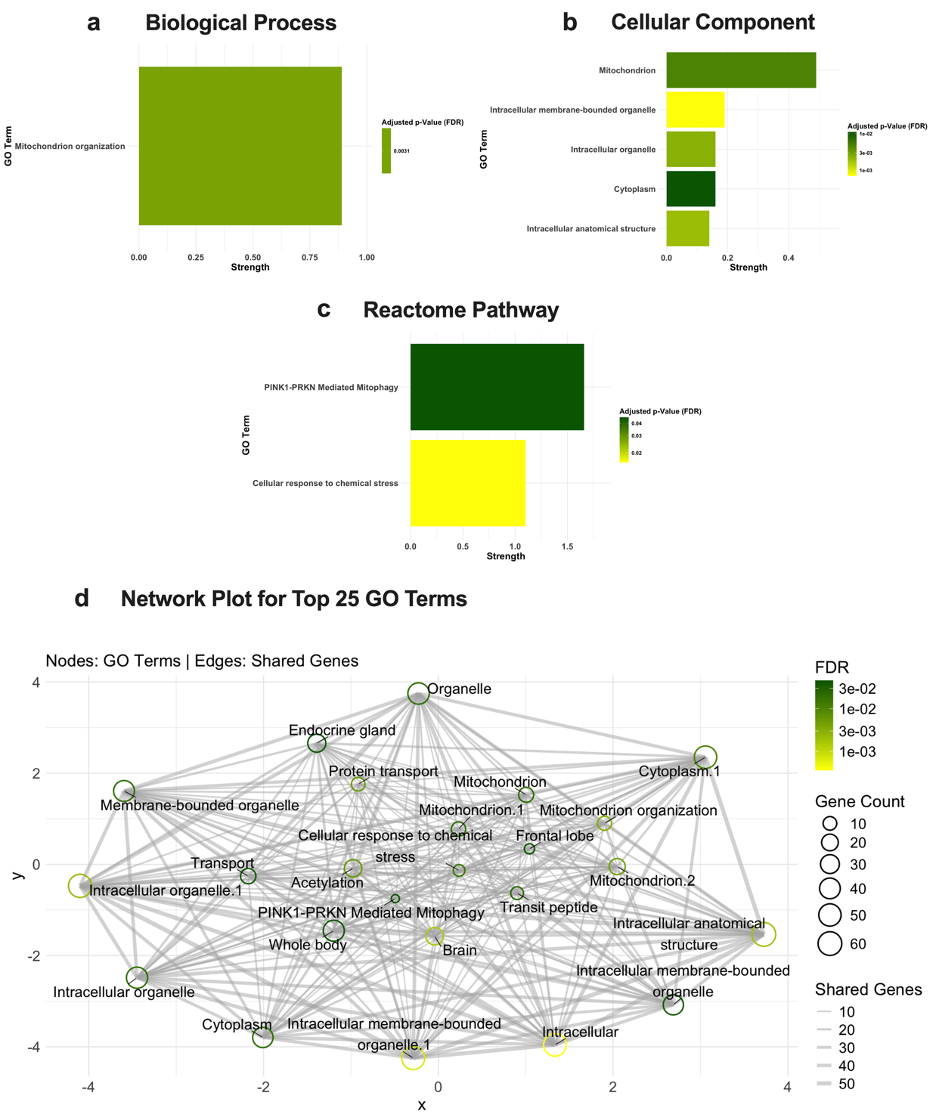


**Supplementary Figure 2: Male Gene Ontology (GO)** Enrichment Analysis for significantly upregulated proteins (p-value <0.05) in male *Negr1^-/-^* mice using STRING analysis. GO term enrichment for (a) Biological Process, (b) Cellular Component and (c) Reactome Pathway. Bar plots show the top enriched GO terms, with the x-axis representing enrichment strength, and bar color indicates the FDR (false discovery rate). (d) displays a network plot of the top 25 GO enrichment terms. Nodes represent GO terms, and edges represent shared genes between terms. Node size corresponds to gene count, edge thickness indicates the number of shared genes, and node color indicates the FDR.


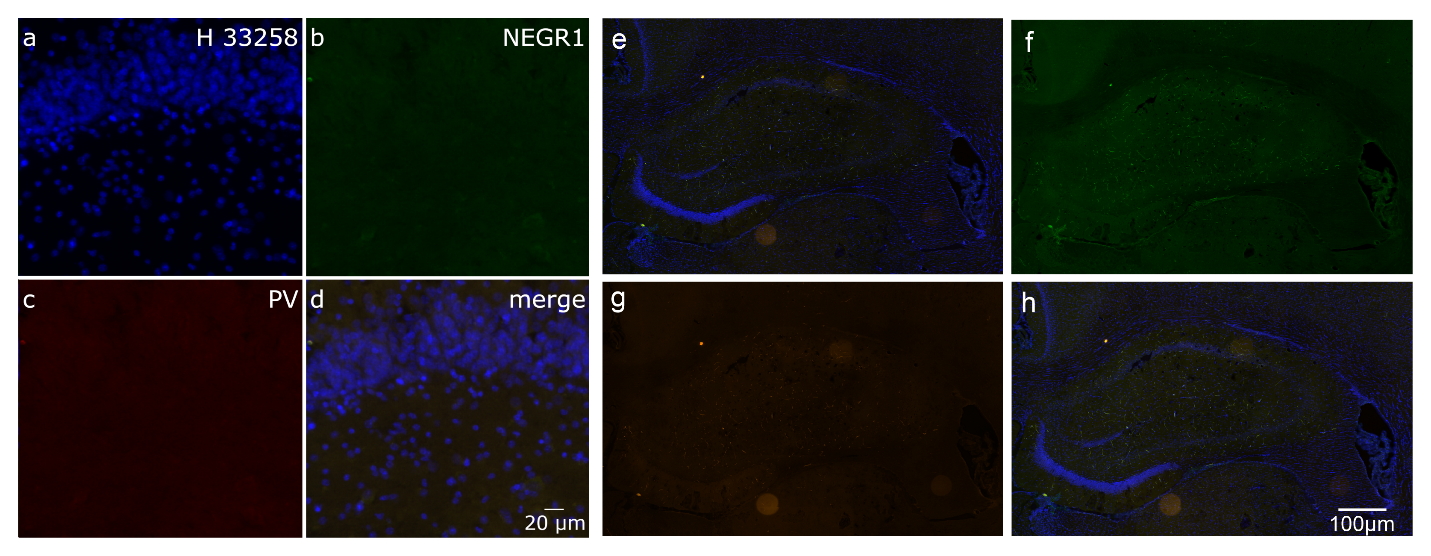


**Supplementary Figure 3:** Negative control immunofluorescence images of human brain tissue (a–d) and rat brain tissue (e–h). Sections were counterstained with Hoechst 33258 to visualize nuclei in blue. Control sections were incubated with secondary antibodies only, matching those used in the NEGR1 (b, f) and PV (c, g) and merge images (d, h) for the staining experiments.
